# In Situ Probing of Electrochemical Hydrogen Evolution Reaction Intermediates: From Single-Crystal Models to Nano-Catalysts

**DOI:** 10.1007/s40820-026-02286-6

**Published:** 2026-07-06

**Authors:** Zhen Zhang, Xing Chen, Jian-Hui Shi, Zhi-Feng He, Daniel Cheung, Gen Huang, Xiaobin Zhong, Jin-Chao Dong

**Affiliations:** 1 School of Energy and Power Engineering, North University of China, Taiyuan, 030051 People’s Republic of China; 2 College of Energy, State Key Laboratory of Physical Chemistry of Solid Surfaces, iChEM, College of Chemistry and Chemical Engineering, Xiamen University, Xiamen, 361005 People’s Republic of China; 3 Innovation Laboratory for Sciences and Technologies of Energy Materials of Fujian Province (IKKEM), Xiamen, 361005 People’s Republic of China

**Keywords:** Hydrogen evolution reaction, Reaction intermediate, Single-crystal electrocatalysis, Nano-electrocatalyst, In situ characterization

## Abstract

Understanding electrochemical hydrogen evolution reaction (HER) mechanisms requires precise identification of key intermediates (H*, OH*, and H_2_O*). While in situ single-crystal studies have provided foundational mechanistic insights into interfacial dynamics and intermediate behavior during the HER, extending these findings to structurally complex nano-catalysts remains challenging. Recent advances in in situ characterization techniques have enabled real-time observation of reaction intermediates, yet a systematic understanding across diverse catalyst architectures remains incomplete. This review assesses HER intermediate research, bridging the gap from model single-crystals to nano-catalysts by: (i) discussing methods for intermediate identification and their roles in elucidating HER mechanisms, (ii) summarizing single-crystal surface modification strategies bridging single-crystal model and nano-catalyst studies, and (iii) highlighting current challenges and proposing future directions for catalyst design and intermediate characterization, offering valuable perspectives for developing advanced HER electrocatalysts.

## Highlights

- Comprehensive evaluation of intermediate identification approaches and their mechanistic significance in the electrochemical hydrogen evolution reaction.
- Systematic analysis of single-crystal surface modification strategies that bridge fundamental models and nano-catalyst studies.
- Current challenges and future directions for efficient hydrogen evolution reaction catalyst design and precise in situ intermediate characterization.

## Graphical abstract

**Figure:**
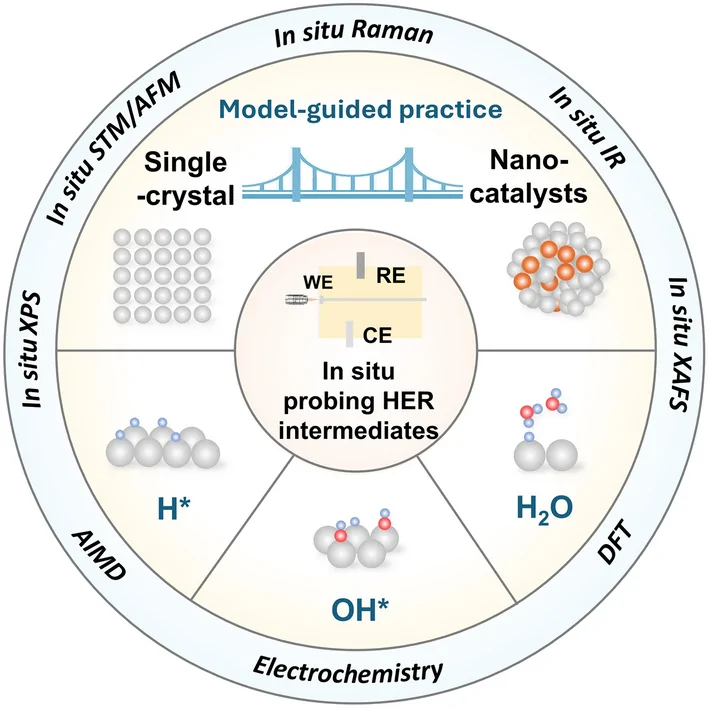

## Introduction

The hydrogen evolution reaction (HER) plays a pivotal role in sustainable energy technologies such as water electrolysis, offering a clean pathway for hydrogen production [1–5]. Fundamentally, the HER efficiency depends critically on the behavior of key intermediates, notably adsorbed hydrogen (H*), hydroxyl (OH*), and interfacial water (H_2_O*), whose identification and dynamic evolution guide catalyst design principles [6–8]. While traditional electrochemical characterization techniques and density functional theory (DFT) simulations provide essential thermodynamic and kinetic parameters, they cannot capture the dynamic evolution of short-lived intermediates due to insufficient spatial and temporal resolution under in situ conditions [9–12].

Recent advances in in situ spectroscopy/microscopy, including in situ surface-enhanced infrared absorption spectroscopy (SEIRAS) [13, 14], surface enhanced Raman spectroscopy (SERS) [15, 16], X-ray absorption fine spectroscopy (XAFS) [17], and electrochemical scanning tunneling microscopy (EC-STM) [18] have enabled real-time observation of electrocatalysis intermediates. Nevertheless, establishing a unified framework linking intermediate properties to catalytic activity spanning well-defined single-crystal models and complex polycrystalline nano-catalysts remains a significant challenge. This gap in understanding primarily arises from the intricate surface structures of practical nano-catalysts, which obscure clear structure–activity relationships [19, 20]. Metal single-crystal electrodes have been instrumental in electrocatalysis research, enabling significant strides in understanding fundamental interfacial phenomena such as the electrical double layer (EDL) structure [20, 21], the impact of electric fields [22, 23], and the adsorption behavior of intermediates or cations/anions on electrode surfaces [24–26]. Single-crystal model systems have provided atomically resolved mechanistic insights into interfacial dynamics and intermediate evolution during the HER. However, no existing review systematically traces how mechanistic insights obtained on well-defined single-crystal model systems translate to structurally complex nano-catalysts under practical operating conditions. Furthermore, the literature lacks a coherent bridge connecting single-crystal studies to nano-catalyst investigations, leaving researchers without clear guidance on technique selection and experimental design.

This review aims to bridge this gap by examining HER research progress from the perspective of intermediate species, spanning single-crystal electrocatalysis to polycrystalline nano-catalysts, with single-crystals as ideal model systems due to their well-defined lattices and pristine surfaces enabling precise structure–activity correlation. Firstly, the research methods of intermediate species on model single-crystals and their important roles in the HER are reviewed. Secondly, the modification strategies of single-crystals are summarized, which is of great significance for studying the surface mechanism of single-crystals and further transitioning to polycrystalline nano-catalysts. Thirdly, examples of the latest research involving intermediates on the surface of nano-catalysts and their important roles in the HER, are summarized. Finally, the challenges and potential solutions faced by single-crystal and polycrystalline nano-catalysts are discussed, and insights are provided for the improvement of future catalyst design and characterization methods. This analysis aims to provide a valuable reference for developing highly active electrocatalysts and advancing the characterization of critical intermediate species.

### Literature Search Scope and Methodology

We systematically searched the Web of Science and Google Scholar databases for peer-reviewed articles published between January 1980 and March 2026. The key search keywords (e.g., in situ, operando, hydrogen evolution reaction, H* intermediate, OH* intermediate, interfacial water, single-crystal, surface modification and intermediate characterization) are provided. The inclusion criteria (peer-reviewed original research and review articles focusing on in situ/operando characterization of HER intermediates) and exclusion criteria (studies on other reactions, ex situ characterizations, and theoretical works without experimental validation) are also clarified.

## Brief Introductions of the HER Mechanism

### HER Mechanism

**Fig. 1:**
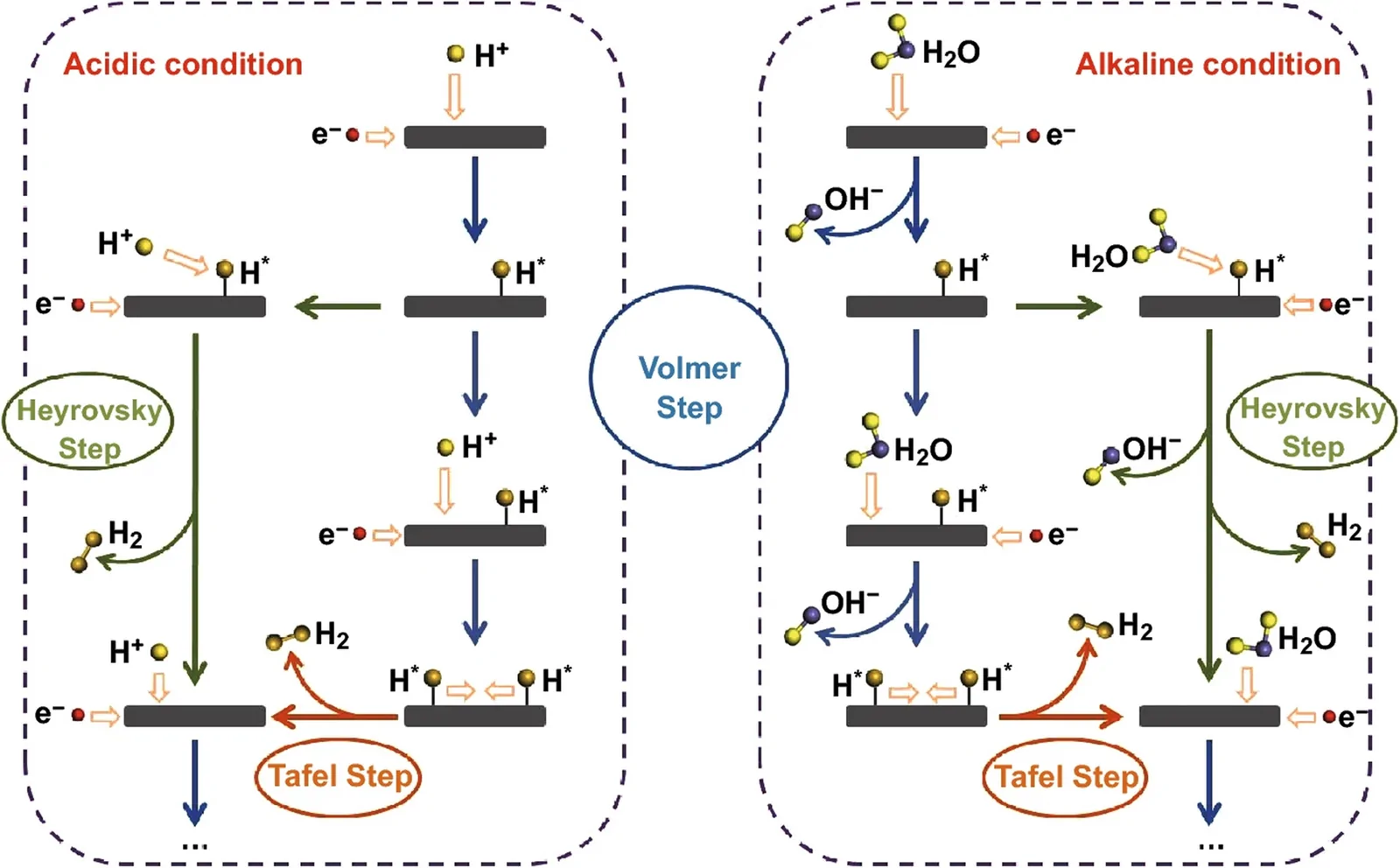
Schematic diagram of HER mechanism [131]

As shown in Fig. 1, the HER proceeds via either the Volmer–Heyrovsky or Volmer–Tafel pathway in both alkaline and acidic media. However, the dominant mechanism remains debated and is highly dependent on the physicochemical properties of the electrode surface [27]. The steps of the HER under alkaline conditions are as follows:

$${\mathrm{H}}_{{2}} {\text{O }} + {\text{ e}}^{ - } \, \to {\text{ OH}}^{ - } \, + {\text{ H}}^* \, \left( {{\mathrm{Volmer}}} \right)$$

$${\mathrm{H}}^* \, + {\text{ H}}_{{2}} {\text{O }} + {\text{ e}}^{ - } \, \to {\text{ OH}}^{ - } \, + {\text{ H}}_{{2}} \left( {{\mathrm{Heyrovsky}}} \right)$$

$${\text{or 2H}}^* \, \to {\text{ H}}_{{2}} \left( {{\mathrm{Tafel}}} \right)$$

The steps of the HER under acidic conditions are as follows:

$${\mathrm{H}}^{ + } + {\mathrm{e}}^{ - } \, + \, * \, \to {\text{ H}}^* \, \left( {{\mathrm{Volmer}}} \right)$$

$${\mathrm{H}}^* \, + {\text{ H}}^{ + } + {\text{ e}}^{ - } \, \to {\text{ H}}_{{2}} \left( {{\mathrm{Heyrovsky}}} \right)$$

$${\text{or 2H}}^* \, \to {\text{ H}}_{{2}} \left( {{\mathrm{Tafel}}} \right)$$

A critical distinction between the acidic and alkaline HER lies in the initial activation step: While the acidic HER involves the direct reduction in hydronium ions (H_3_O⁺), the alkaline HER requires the prior dissociation of a water molecule. This introduces significant additional kinetic barriers, primarily associated with water dissociation and the subsequent hydroxide (OH⁻) desorption. Consequently, alkaline HER generally exhibits substantially slower reaction kinetics and greater mechanistic complexity compared to its acidic counterpart [9, 28–32]. Efficient alkaline HER catalysis is dependent on several critical requirements. First, the catalyst must possess effective water activation and OH⁻ desorption capability. Second, proton supply efficiency is essential: The rate of H_2_O dissociation directly governs the available proton concentration, thus dictating the overall HER kinetics. Third, an optimal hydrogen adsorption free energy (ΔG_H*_) is required: The binding strength of H* on the catalyst surface must be neither too strong nor too weak to facilitate both its formation and recombination/desorption as H_2_.

This mechanistic understanding highlights the need for tailored catalyst design to address the distinct challenges of the alkaline HER, particularly in facilitating water dissociation and intermediate stabilization. To gain such fundamental insights into the behavior of these critical intermediates (H*, OH*, H_2_O*), studies on well-defined single-crystal surfaces are indispensable.

### Key Intermediates in the HER

#### Hydrogen Bond Energy (HBE) Theory (H*)

The hydrogen binding energy (HBE) theory serves as a foundational framework in electrocatalysis, utilizing the metal-hydrogen bond energy as a central descriptor to establish a thermodynamic correlation between a catalyst’s intrinsic activity and its hydrogen adsorption behavior. While this theory has been widely adopted for interpreting HER trends, several key challenges and complexities have emerged when applying it to real electrochemical systems, particularly under varying pH conditions.

A fundamental concept in the electrochemistry of HER is the distinction between underpotential deposition hydrogen (UPD_H_) and overpotential deposition hydrogen (OPD_H_). UPD_H_ refers to hydrogen atoms adsorbed onto the electrode surface at potentials positive of the reversible hydrogen electrode (RHE) potential, typically in the range of approximately 0.05 to 0.40 V vs. RHE on polycrystalline Pt [33, 34]. UPD_H_ species are generally considered spectator species and are not directly involved in the HER pathway at significant overpotentials. In contrast, OPD_H_ refers to hydrogen atoms deposited at potentials negative of the RHE potential, typically below 0 V vs. RHE, where the HER becomes kinetically favorable. OPD_H_ species are generated via the Volmer step, and their subsequent combination via the Tafel or Heyrovsky steps leads to HER. Experimentally, UPD_H_ is readily observed as characteristic adsorption/desorption peaks in cyclic voltammograms of clean Pt electrodes in acidic/alkaline media, while OPD_H_ is not directly observable as distinct voltammetric features. However, in situ spectroscopic techniques such as SEIRAS and SERS have enabled direct detection of OPD_H_ species by distinguishing their vibrational signatures, which differ subtly due to variations in adsorption site geometry and coverage-dependent dipole coupling. A clear differentiation between these two hydrogen adsorption regimes is therefore essential for correctly interpreting electrochemical and spectroscopic data in HER research.

Notable experimental observations present apparent discrepancies with simplified HBE-based predictions. For instance, the underpotential deposition hydrogen (UPD_H_) peak position on Pt(111) surfaces shows no significant pH dependence, a finding that contrasts with the marked differences in Pt(111) activity observed between acidic and alkaline environments [35]. Furthermore, adsorption energies for overpotential deposition hydrogen (OPD_H_) obtained from spectroscopic techniques often differ substantially from those derived from electrochemical UPD_H_ analyses [11, 29]. The interfacial complexity of electrochemical environments, especially in alkaline media where cations modulate the electrical double layer structure, further complicates the picture by influencing water dissociation kinetics and OPD_H_ adsorption thermodynamics [29, 31, 36, 37]. Additionally, high hydrogen coverage can induce surface reconstruction or H–H interactions, leading to substantial deviations from HBE values predicted by low-coverage models [29, 30].

Thus, while the HBE theory provides a valuable thermodynamic starting point, its descriptive power for operational catalytic interfaces is inherently constrained by the simplified nature of its core assumptions relative to the multifaceted reality of electrochemical systems. A more complete mechanistic understanding of pH-dependent HER activity likely requires the integration of HBE with dynamic interfacial properties, such as solvent reorganization, adsorbate interactions, and potential-dependent structural changes, to bridge the gap between thermodynamic descriptors and kinetic outcomes.

#### Bifunctional Theory (OH*)

The bifunctional theory emphasizes the role of OH* in H_2_O dissociation, proposing a synergistic mechanism where distinct active sites independently facilitate proton adsorption and desorption, overcoming the limitations of single-site catalysis. While the theory offers a valuable conceptual framework for interpreting alkaline HER activity, several aspects merit further consideration when extending it across different electrochemical environments.

First, the initial step of the HER differs fundamentally between acidic and alkaline conditions, direct proton reduction in acid versus water dissociation in alkali. This suggests that the role and even the necessity of bifunctional sites involving OH* may vary considerably with pH and could be less pronounced in acidic environments [38]. Second, under certain conditions, OH* may act as a spectator species rather than active participants, potentially blocking active sites for hydrogen adsorption and thereby inhibiting the HER [39, 40]. Third, the oxophilic component of bifunctional catalysts may modulate activity primarily through interfacial electron transfer effects, rather than solely through the optimization of OH* binding energy as often assumed [30, 41]. Thus, while the bifunctional theory provides a critical conceptual framework for understanding the alkaline HER, its predictive capacity is limited by its representation of the electrochemical interface. A comprehensive description requires the explicit integration of dynamic factors such as interfacial electric fields, potential-dependent solvation effects, and adsorbate coverage phenomena to fully explain pH-dependent catalytic mechanisms.

#### ***H***_***2***_O* Structure Theory (H_***2***_***O)***

In contrast to the HBE and bifunctional theories, which primarily rely on thermodynamic adsorption energies of key intermediates (H* or OH*), the interfacial H_2_O* structure theory focuses on the dynamic structural and orientational properties of water molecules within the EDL and their profound influence on the HER/HOR kinetics. Despite growing attention, current understanding of how water structure governs kinetics remains fragmented. For instance, Koper et al. proposed that in alkaline conditions, the large potential difference between the pH and the point of zero charge (PZC) stabilizes rigid H_2_O* structures, which act as key kinetic barriers for the HER [9]. Other studies, however, report seemingly contrasting observations: Some indicate that intact hydrogen‑bonding networks may promote the HER [28, 42], whereas others propose that disrupted networks with more free water molecules are beneficial [43, 44].

These divergent findings underscore the immense challenge in studying interfacial H_2_O*, stemming from its anomalous physical properties, structural complexity, and dynamic response to the local electrochemical environment. While emerging in situ techniques continue to provide new insights into the dynamic nature of water at interfaces, they simultaneously reveal new puzzles to be solved.

### Intermediate Species Characterization Techniques

**Fig. 2:**
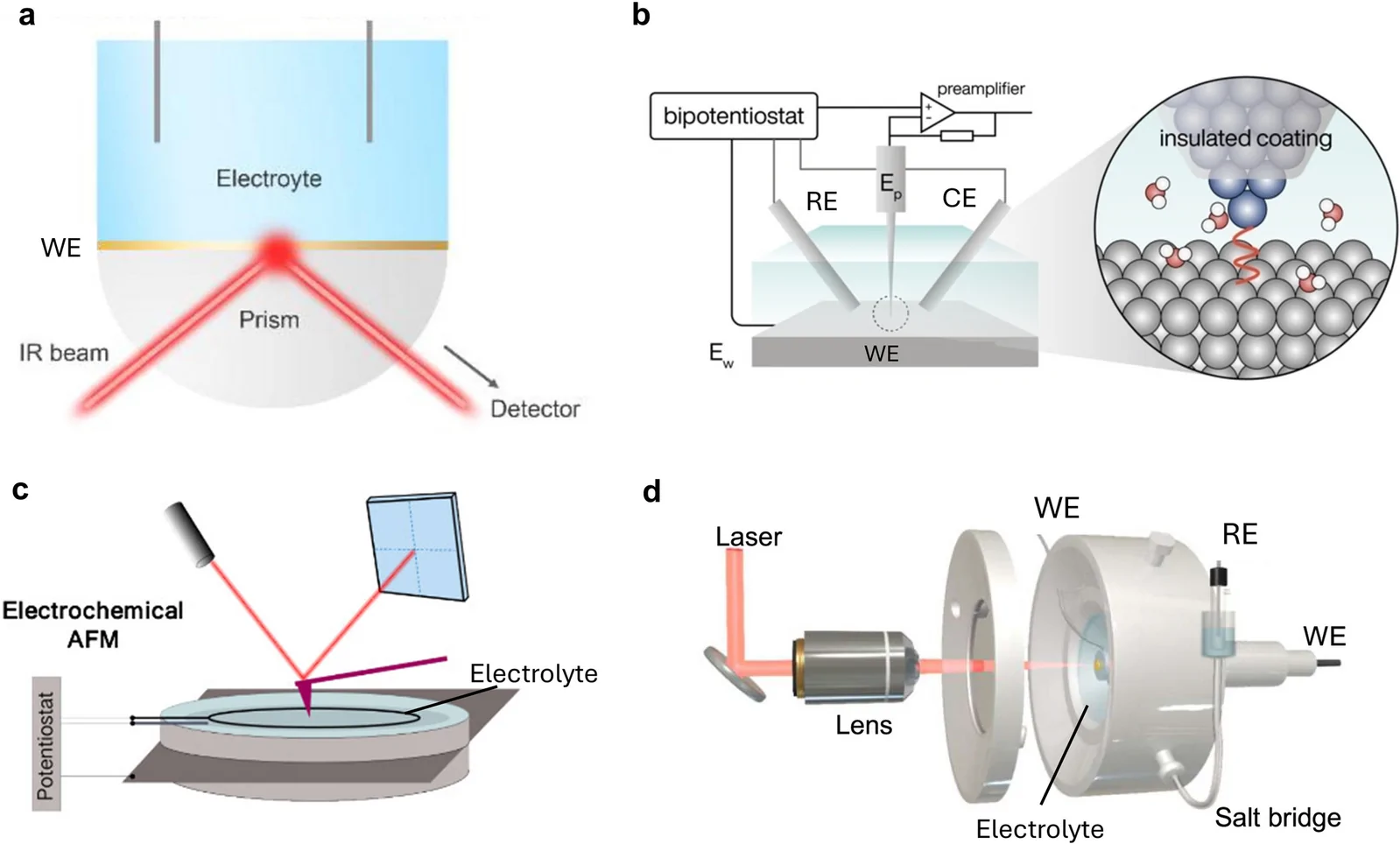
Schematic illustrations depicting the fundamental principles and corresponding instrumentation configurations of various in situ characterization techniques: **a** EC-SEIRAS [132], **b** EC-STM [133], **c** EC-AFM [134] and **d** EC-SERS [98]. Working electrode (WE), reference electrode (RE) and counter electrode (CE)

A brief introduction to the principles of the key in situ/operando characterization techniques is provided, with corresponding illustrations shown in Fig. 2. Electrochemical (EC) SEIRAS exploits the enhanced infrared absorption of molecules adsorbed on nanostructured metal surfaces (e.g., Au) to identify vibrational fingerprints of reaction intermediates (Fig. 2a). EC-STM measures tunneling current between a conductive tip and the electrode surface under potential control, providing atomic-resolution topographic imaging of adsorbed intermediates and surface structures (Fig. 2b). EC atomic force microscope (AFM) uses a sharp tip to scan the electrode surface while monitoring force interactions, delivering topographic and adsorption information of intermediate-covered surfaces under electrochemical conditions (Fig. 2c). EC-SERS relies on localized surface plasmon resonance to greatly amplify Raman scattering from adsorbed species, enabling molecular-level detection of low-concentration intermediates (Fig. 2d). Moreover, electrochemical CV can probe reaction intermediates by recording current responses as a function of applied potential, capturing redox features associated with adsorbed species.

To provide readers with a systematic and practical guide for selecting appropriate in situ/operando characterization techniques for HER intermediate studies, we have compiled Table 1, which compares the major techniques discussed in this review. The comparison is structured across several critical dimensions, including the types of probe species or intermediates detectable, key limitations, and recommended complementary techniques for data integration. Both electrochemical methods (CV) and spectroscopic/microscopic techniques (EC-SEIRAS, EC-SERS/SHINERS, EC-STM, EC-AFM, etc.) are included to highlight their respective strengths and inherent limitations.

**Table 1:** Comparison of in situ/operando characterization techniques for HER intermediate analysis

Technique	Probe intermediates	Sensitivity	Key limitations	Complementary techniques
SEIRAS	H*, H_2_O, adsorbed anions	High for sub-monolayer	Au film required for enhancement; ensemble averaged signal	SERS/SHINERS (for Raman-active species, especially the testing of low-wavenumber oxygen-containing intermediates), EC-STM (for spatial correlation)
SERS	H*, OH*, H_2_O, intermediates, surface species	Sub-monolayer adsorbed species and single-molecule level	Limited to Au, Ag, Cu (strong enhancement) Potential influence of electronic structure from enhanced substrate;	SEIRAS (for IR-active species)
STM	H*, OH*, H_2_O, surface species	High for structural features; cannot directly identify chemical identity	Cannot directly identify chemical species; requires atomically flat surfaces; slow imaging limits observation of fast dynamics	EC-AFM (for non-conductive or soft surfaces), SERS/SHINERS (for chemical identification), DFT (for structural assignment)
AFM	Surface morphology, volume changes, bubble nucleation, catalyst restructuring	High for topography; no chemical specificity	No direct chemical identification; tip-surface interaction may disturb the sample	EC-STM (for higher resolution on conductive surfaces), SERS (for Raman-active species), SEIRAS (for molecular information)
CV	UPD_H_, OH* adsorption/desorption	High for surface coverage	Cannot directly identify specific intermediates; the identification of intermediates poses considerable difficulty	SEIRAS/SERS (to assign CV features to specific species), EC-STM (to correlate with surface structure)

## HER on Model Single-Crystal Surface

### HER Intermediates on Single-Crystal Surfaces

Single-crystals possess a continuous and unbroken crystal lattice with a uniform, well-defined atomic arrangement throughout the entire structure. Their importance lies in providing pristine, well-characterized surfaces with specific crystallographic orientations (e.g., (111), (100)), enabling the precise correlation between atomic-level surface structure, intermediates, and electrocatalytic activity/mechanism.

Intermediate species on single-crystals are detected using a variety of methods, including electrochemistry, SEIRAS, SERS, STM, and theoretical calculations.

#### Hydrogen Adsorption (H*)

Hydrogen adsorption manifests as two distinct states: UPD_H_, occurring above the equilibrium potential, and OPD_H_, which serves as the key reactive intermediate in the HER [45, 46], both of which are key topics in energy catalysis. The characterization of H* behavior on metal single-crystal surfaces relies heavily on advanced instrumental techniques, which provide crucial experimental evidence for understanding the HBE.

**Fig. 3:**
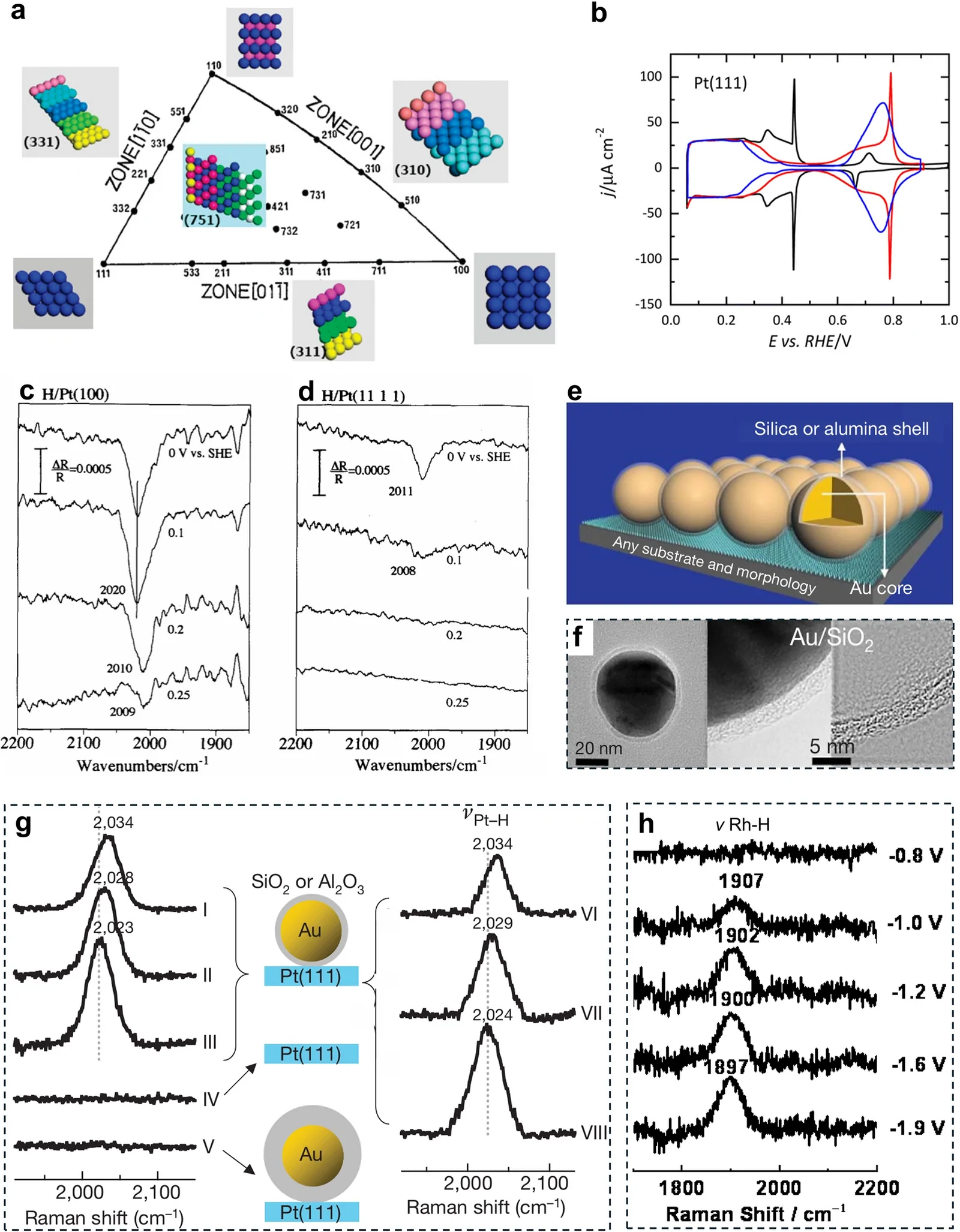
**a** Unit stereographic triangle of FCC metal single-crystal and models of surface atomic arrangement. Reproduced with permission [135]. American Chemical Society The behavior of H* on single-crystal. CVs of **b** Pt(111). Reproduced with permission [68]. Copyright 2024, The Royal Society of Chemistry. IRAS spectra of H* on **c** Pt(100) and **d** Pt(111) in 0.5 M H_2_SO_4_. Reproduced with permission [56]. Copyright 1994, Elsevier. **e** Schematic diagram of SHINERS. **f** TEM images of Au@SiO_2_. **g** SHINERS spectra on a Pt(111) surface. Reproduced with permission [16]. Copyright 2010, Springer Nature. **h** SHINERS spectra on a Rh(111) surface. Reproduced with permission [62]. Copyright 2011, The Royal Society of Chemistry

To intuitively illustrate the distinct atomic configurations of these crystalline surfaces, Fig. 3a presents schematic diagrams of the typical Pt(hkl) single-crystal models discussed in these studies. In the realm of electrochemical characterization, cyclic voltammetry (CV) serves as the most fundamental electrochemical technique. Seminal work by Frumkin et al. on polycrystalline Pt revealed symmetric voltammetric peaks corresponding to H* adsorption/desorption [47]. They discovered that H* on the surface of polycrystalline Pt gives rise to characteristic voltammetric peaks with a high degree of symmetry. These peaks not only determine the interfacial cleanliness but also enable precise quantification of the electrochemically active surface area (ECSA) [48–50]. Over subsequent decades, researchers have conducted systematic studies on the UPD_H_ behavior of Pt single-crystal in various electrolytes (Fig. 3b) [51, 52]. These studies have demonstrated that the CV curves of different single-crystal surfaces and diverse types of electrolytes exhibit pronounced disparities, providing ample evidence of the dependence of H* behavior on the atomic arrangement structure of the single-crystal surface and the composition of interfacial EDL. In electrolytes containing specifically adsorbed anions, the co-adsorption of anions renders the voltammetric response extremely intricate, posing a serious challenge to the precise analysis of experimental data. Lasia, through theoretical calculations of the UPD_H_ polarization curves of low-index single-crystal Pt(hkl) in HClO_4_ and H_2_SO_4_, has determined that the surface coverage exhibits a linear dependence on the adsorbate interaction energy, following a Frumkin-type isotherm. [53]. Meanwhile, Markovic et al. found that there exists a strong lateral repulsive force in the H* adsorption layer on Pt(111). The UPD_H_ adsorption energy linearly decreases, indicating that UPD_H_ possesses remarkable structural sensitivity [54, 55]. It is worth emphasizing that the calculations indicate that even at a potential of > 0.05 V, the coverage (θ) of Pt(111) only reaches 66% and a potential of −0.1 V is required to achieve complete monolayer hydrogen adsorption. The limited H* coverage observed at potentials between 0.05 and 0.4 V precludes a robust assessment of the HBE theory, as there are substantial differences when the single-crystal surface is fully covered with H*. Therefore, a rigorous application of HBE theory must account for these coverage-dependent and structurally coupled effects.

While electrochemistry provides indirect insights, direct spectroscopic identification is essential for mechanistic understanding. As early as the last century, in situ infrared reflection absorption spectroscopy (IRRAS) studies on Pt(hkl) surfaces revealed Pt–H stretching vibrations (1990–2080 cm⁻^1^) for terminal UPD_H_ on Pt(100) and Pt(110) (Fig. 3c, d) [56]. The absence of such bands on well-ordered Pt(111) indicates the dependence of H* adsorption configuration on the crystallographic orientation. Notably, well-oriented Pt(111) electrodes exhibited no discernible absorption for terminal H* under similar conditions, suggesting that terminal H* is highly sensitive to surface crystallographic orientation. Nevertheless, direct spectral detection of UPD_H_ remains challenging due to its extremely low Raman scattering cross-section, likely arising from symmetric adsorption geometries and dipole-inactive vibrational modes [55, 57–59]. Despite the extensive research on UPD_H_ behavior through electrochemical methods, direct spectral detection still confronts significant technical challenges owing to its special structural features. Even with the aid of SERS and other spectra with signal enhancement, the existence of UPD_H_ has not been directly observed. In recent years, limited progress has been achieved in the research on the real intermediate species OPD_H_ in the HER [60].

R. J. Nichols et al. observed OPD_H_ (2090 cm⁻^1^) in the HER potential range on the surfaces of polycrystalline Pt and single-crystal Pt(111) using in situ IRRAS, with confirmation via the characteristic isotopic shift to ~ 1500 cm^−1^ in D_2_O electrolyte [61]. Subsequent advances in surface-enhanced Raman spectroscopy (SERS) enabled Tian et al. to observe top-site OPD_H_ on polycrystalline Pt. This culminated in the application of shell-isolated nanoparticle-enhanced Raman spectroscopy (SHINERS) to Pt(111). SHINERS, developed by Tian, addresses a limitation of conventional SERS: the requirement for atomically rough or nanostructured metal substrates (e.g., Au, Ag, Cu) to achieve plasmonic enhancement, which precludes direct application to well-defined, atomically flat single-crystal surfaces such as Pt(111) or Rh(111) [16]. In SHINERS, Au nanoparticles are encapsulated by an ultrathin, pinhole-free silica or alumina shell (~ 2 nm thick). This inert shell serves two critical functions: It prevents direct contact and charge transfer between the plasmonic core and the catalytically active surface, thereby avoiding perturbation of the surface intrinsic electrochemical properties; it maintains a uniform, tunable gap between the nanoparticle and the electrode surface, generating intense electromagnetic field enhancement (hot spots) that amplifies the Raman signal from adsorbed species (Fig. 3e). The SHINERS result reveals a broad Pt–H stretch at 2023 cm^−1^ within the HER potential window (Fig. 3f) [16]. This approach was later extended to identify the Rh-H vibration on Rh(111) at 1907 cm^−1^ [62]. Additionally, methodologies exist (e.g., high-resolution electron energy-loss spectroscopy (HREELS)) for investigating hydrogen adsorption on Pt single-crystal surfaces under ultra-high vacuum (UHV) conditions [63, 64]. However, constrained by the UHV environment, these methods exhibit significant discrepancies from the conditions of practical electrocatalytic systems, resulting in extremely limited applications within the electrocatalysis field at present.

In summary, the characterization of H* on metal single-crystal surfaces has advanced from indirect electrochemical methods to direct spectroscopic identification, yet challenges persist. CV has revealed the structural sensitivity of UPD_H_. However, direct spectroscopic detection of UPD_H_ remains elusive due to its low Raman scattering cross-section and dipole-inactive modes, even with SERS. In contrast, OPD_H_, the key HER intermediate, has been successfully detected using in situ spectroscopy on atomically flat surfaces. Collectively, while significant progress has been made in probing OPD_H_, the detection of UPD_H_ remains an open challenge.

#### OH Adsorption (OH*)

The role of OH* in the HER is complex and critical. While traditionally central to bifunctional mechanisms in alkaline media, its exact function (as a direct participant, a spectator, or an electronic modifier) remains a subject of active debate [65–67]. Advanced characterization techniques have been essential in probing its adsorption behavior and elucidating these competing perspectives.

The oxophilicity (or OH⁻ adsorption strength) of the electrode could evaluated from the CV response in the potential window of 0–0.8 V vs. RHE. As shown in Fig. 3b, there is still disagreement regarding paired redox peaks near 0.8 V RHE in the CV of Pt(111) [68]. However, the overall trend suggests that they are related to the adsorption of OH*. Watanabe et al. attributed these peaks to the coexistence of HOH* and OH* species through electrochemical X-ray photoelectron spectroscopy (EC-XPS) [69]. Based on the results of electrochemical and theoretical calculations, the latest research by Chen et al. attributed these peaks to the varying Pt-H_2_O* distance under different OH* coverages [70]. Feliu et al. believed that this characteristic reflects the differential dissociation behavior of water molecules with different structures [71]. Rinaldo et al. proposed that this is caused by the adsorption of hydroxide ions at two different surface sites [72]. Koper et al. believed that these two characteristic peaks reflect the type of interaction of the adsorbed species rather than two different adsorption modes [73]. K. Bedürftig et al. confirmed through HREEL and STM images that OH* exists at the top sites of Pt atoms in a single adsorption state and its molecular axis is tilted relative to the surface normal. In this orientation, OH* forms hydrogen bonds with adjacent hydroxyl molecules [74].

**Fig. 4:**
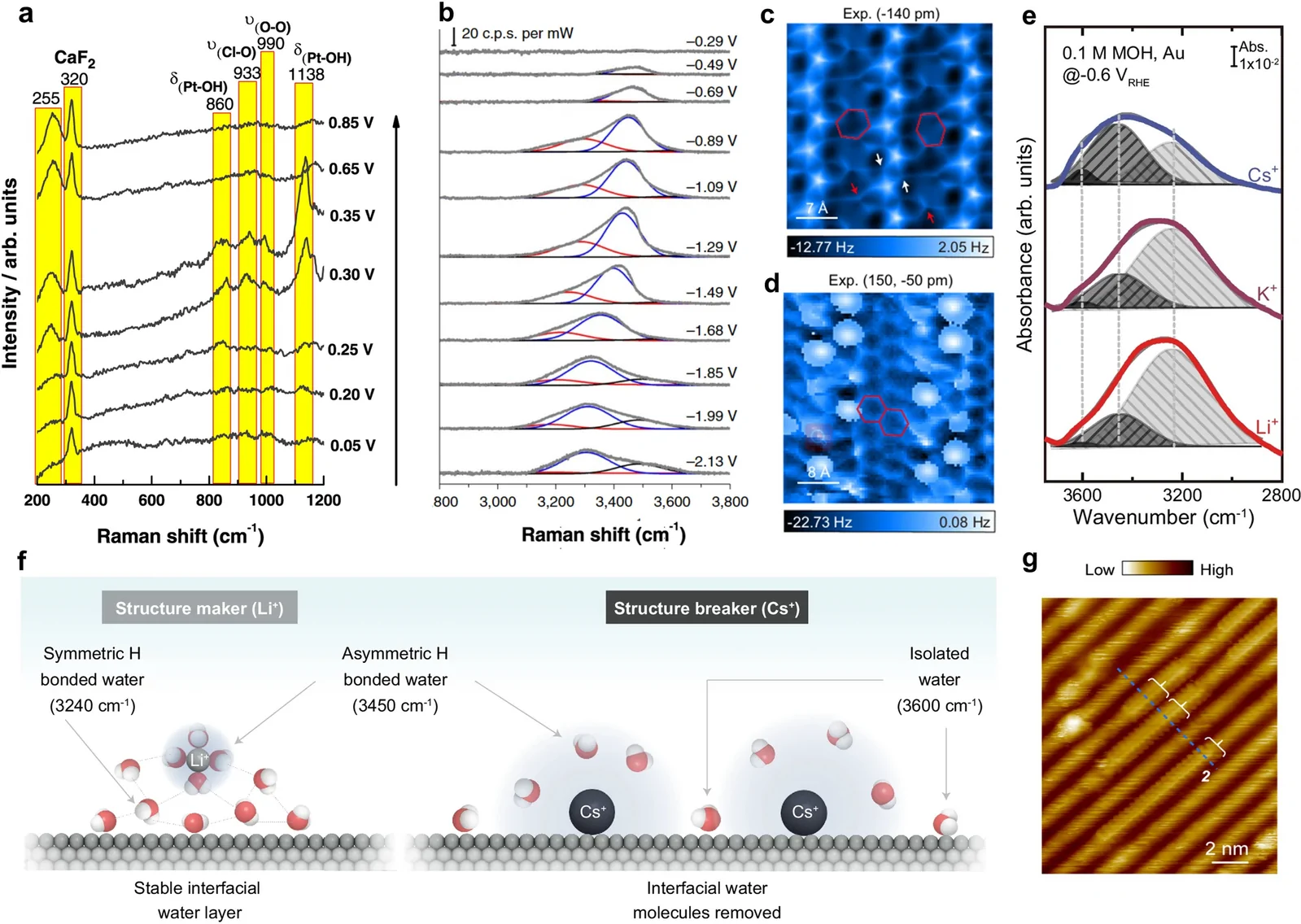
Behavior of OH* and H_2_O*. **a** In situ SHINERS of the Pt(311) electrode measured at varying potentials. All experiments were performed in an Ar-purged 0.1 M HClO_4_ electrolyte. Reproduced with permission [76]. Copyright 2022, Springer Nature. **b** In situ Raman at the Au(111) measured in 0.1 M Na_2_SO_4_ electrolyte solution. Potential-dependent evolution of the hydrogen-bond network of H_2_O*. Reproduced with permission [80]. Copyright 2019, Springer Nature. **c, d** Constant-height AFM images and corresponding structural model of 2D ion-water structure on the Au(111). **e** OH stretching features of in situ SEIRAS spectra in 0.1 M LiOH, KOH, and CsOH. **f** Schematic illustration of the interfacial behavior of Li⁺, Cs⁺, and H_2_O*[42]. Copyright 2024, Springer Nature. **g** Atomically resolved STM image of a bare anatase TiO_2_ (001)-(1 × 4) surface and water-adsorbed surface. The fuzzy lines indicate the moving H_2_O molecules. Reproduced with permission [81]. Copyright 2022, American Chemical Society

Interestingly, on high-index Pt(hkl), OH* exists even within the potential range of UPD_H_ (0–0.4 V vs. RHE) [75]. Feliu et al. found in their study of the Pt (311) crystal plane that OH* can be adsorbed at a low potential on low-coordination Pt atoms. By combining CO displacement experiments with alternating current voltammetry, they not only detected the presence of OH* on the Pt surface but also calculated its coverage to be approximately 0.24. More notably, they directly observed the OH* species on the surface of Pt (311) for the first time using the SHINERS technique (Fig. 4a). The Raman peak at 860 cm^−1^ is assigned to the bending vibration of Pt-OH caused by the adsorption of OH* at the Pt(100) step sites, while the peak at 1138 cm⁻^1^ corresponds to the vibrational mode of OH* near the oxygen-bridged Pt sites[76].

While some subsequent literature reports have presented negative effects of OH*: (i) OH* blocks the adsorption of the active intermediate H*: Intikhab et al. believed that OH* is adsorbed on the active sites of Pt, which reduces the adsorption of H on the surface sites instead of directly participating in the Volmer step, thus reducing the hydrogen evolution activity [77]. (ii) Electronic structure modification: Nash et al., by electrochemically leaching Ru from the Pt-Ru alloy, observed that only the subsurface Ru atoms, rather than the surface Ru atoms, are important for maintaining high HER/HOR performance, which further challenges the bifunctional mechanism [78]. This leads to a dilemma, as simply coating the Pt surface with a small amount of Ru can still significantly improve the HER performance.

In summary, the role of OH* in the HER remains complex and debated, with evidence supporting its function as a direct participant, a spectator, or an electronic modifier depending on the specific conditions. However, negative effects of OH* have also been reported, including blocking of active H* sites and acting as an electronic modifier via subsurface atoms. These contrasting observations underscore that the role of OH* is highly sensitive to surface structure, potential and electrolyte conditions.

#### ***Interfacial Water Structure (H***_***2***_O*)

Electrocatalytic reactions occur at the solid–liquid interface within an aqueous liquid electrolyte, making the structure and dynamics of interfacial water molecules (H_2_O*) a critical, yet complex, factor in determining reaction kinetics. The orientation of H_2_O* in the EDL and its hydrogen-bonding network directly influence charge transfer, ion transport, and intermediate stabilization [32, 79]. However, a precise, potential-dependent mapping of H_2_O* configurations remains a significant challenge.

A foundational perspective was provided by Koper et al., who linked sluggish alkaline HER kinetics to interfacial water structure. They proposed that in alkaline media, the large potential difference between the operating HER potential and the PZC generates a strong interfacial field. This field promotes a rigid, ordered H_2_O* structure that impedes necessary water reorientation and OH^−^ transport, thereby increasing the energy barrier of the initial Volmer step [9]. Impedance spectroscopy confirms that the rate of H* decreases as the pH increases, and the charge transfer resistance (R_ct_) increases, confirming that the H_2_O* structure has a strong influence on the kinetic sluggishness, providing a new perspective on the alkaline HER kinetics.

SERS and SEIRAS serve as core characterization techniques. By detecting the O–H stretching vibrational modes in the range of 3200–3700 cm⁻^1^ (3220 cm⁻^1^ corresponds to a strong hydrogen bond network, and 3400 cm⁻^1^ and 3600 cm⁻^1^ correspond to weak hydrogen bonds), the degree of order of the H_2_O* hydrogen bond network can be quantitatively analyzed. Li et al. successfully recorded the Raman signal of H_2_O* on Au (111) (Fig. 4b). They observed two orientations of H_2_O* during the HER, noting that the H_2_O* transitioned from a “parallel” structure to a “one-H-down” structure and then to a “two-H-down” structure with a negative shift of potential. Furthermore, employing AIMD, they simulated three orientations of H_2_O* and the corresponding hydrogen bond numbers in the EDL under different potentials, which aligned well with the experimental data, providing further insight into the atomic structure of the electric double layer. Such findings provide valuable insights for exploring the detailed molecular arrangement within the EDL [80].

Jiang et al. directly observed the structures formed by alkali metal ions (Li⁺, K⁺, Cs⁺) and H_2_O* on the surface of Au(111) using a modulated AFM[42]. Experimental results from high-resolution AFM indicate that alkali metal ions distinctly affect the ordering of the hydrogen bond network of H_2_O* (Fig. 4c, d). It was found that Li⁺ promotes ordering of the hydrogen bond network of H_2_O* (Structure maker), while Cs^+^ causes significant distortion (Structure breaker). To further explore the influence of cations on the hydrogen bond network at the electrolyte/electrode interface, researchers performed in situ SEIRAS experiments (Fig. 4e). By analyzing the vibrational modes and intensities of the OH vibration peaks of water molecules, SEIRAS provides information on the ordering of the surface hydrogen bond network. Experiments revealed a more ordered hydrogen bond network of H_2_O* at the Li⁺ electrolyte/electrode interface (the peak at 3240 cm⁻^1^ is stronger than those at 3450 and 3600 cm⁻^1^). In contrast, Cs^+^ promotes the formation of weakly hydrogen-bonded H_2_O* (at 3600 cm^−1^), distorting the network and forming a disordered hydrogen-bonding environment. This disordered network hinders proton transfer, exhibiting an inhibitory effect that ultimately lowers reaction efficiency (Fig. 4f). These SEIRAS findings are consistent with those of the AFM experiments.

Complementary to AFM, STM has emerged as a powerful tool for molecular-scale water visualization. Wang et al. demonstrated through STM that specific interfacial hydrogen-bonding configurations can promote H_2_O dehydrogenation at sub-monolayer (< 1 ML) coverages (Fig. 4g). However, since weak hydrogen bonds are particularly susceptible to STM tip perturbation, complementary spectroscopic techniques are required to unambiguously characterize their role. In situ ultraviolet photoelectron spectra (UPS) reveal that step-edge water dissociation is activated by photoexcited holes, which oxidize adsorbed water species at step sites to generate both OH⁻ species and radical-like OH* intermediates. Furthermore, the interfacial hydrogen-bonding network plays two crucial roles: It significantly reduces the dissociation energy barrier and facilitates coupled hole–proton transfer processes [81].

Theoretical simulations provide strong supporting evidence for the study of interface water structure. Cheng et al. simulated the structure of the Pt(111)/aqueous electrolyte interface under different potentials. Their analysis revealed that the coverage of chemisorbed water on Pt(111) follows the Frumkin adsorption isotherm with applied potential. Furthermore, they theoretically demonstrated that the potential-dependent change in chemisorbed water coverage contributes to negative capacitance behavior at the electrochemical interface, enhancing our understanding beyond the classical electric double layer model [82]. The same group further extended such research to the high-index single-crystals. The Pt(211)/water interface was studied based on the machine learning molecular dynamics method. They identified five distinct adsorption states for water molecules (including physisorbed and chemisorbed) and further characterized three specific configurations of hydrogen-bonded water dimers. These identified adsorption states and dimer configurations largely govern the anisotropy in the dynamics of adsorbed water molecules [83]. The theoretical calculation research conducted by Zhu et al. emphasized the role of cations in the EDL. Using AIMD, they found that at the Pt(111)/water interface, the hydration of cations and their interaction with the H_2_O* structure jointly determine the connectivity of the hydrogen bond network in the electrical double layer [84].

In summary, the structure and dynamics of interfacial water play a critical role in determining HER kinetics. A large potential difference between the HER potential and the PZC in alkaline system, which promotes a rigid H_2_O network that impedes alkaline HER. Complementary AFM and SEIRAS studies demonstrate that Li⁺ acts as a structure maker, promoting an ordered hydrogen-bond network that facilitates proton transfer, while Cs⁺ acts as a structure breaker, inducing a disordered network that hinders HER kinetics. These findings underscore that H_2_O structure is highly sensitive to pH, potential and cation identity, and the precise control is essential for optimizing HER performance.

### Research on Surface Modification of Single-Crystals

Heterostructured interfacial catalysts represent a promising class of materials that optimize catalytic performance by engineering synergistic interfaces [85]. By introducing new components to construct the heterogeneous interface, the electronic structure of the catalyst can be optimized, the adsorption energy and adsorption mode of intermediate species can be adjusted, and the local environment of the catalytic interface can be modified, so as to obtain the desired catalytic effect that is difficult to achieve with a single component catalyst [85, 86]. Within single-crystal electrochemistry, surface modification represents a crucial research direction. This approach influences the catalytic system in two primary ways: i) by altering the geometric structure of the single-crystal surface and ii) by modifying the local electronic structure. In addition, surface-modified species can serve as active catalytic sites in electrochemical reactions, assisting single-crystals in facilitating certain electrocatalytic processes.

#### Interface Effect of Single-Crystal Modification

##### Spillover Effect

The catalytic mechanism involves the dynamic transformation of active centers, where reactive intermediates migrate via spillover to adjacent sites for subsequent reduction steps [87, 88]. This spillover effect presents a new challenge in comprehensively studying the mechanisms underlying electrocatalytic reactions. A thorough understanding of spillover is crucial for solving catalytic mechanisms and is a prerequisite for the rational design of more efficient catalysts. This effect will be discussed in detail later in the context of characterizing hydrogen species on catalysts.

##### Charge Transfer and Synergistic Effects

The enhanced activity observed at heterogeneous interfaces can be attributed to electron redistribution arising from the difference in Fermi energy levels between the single-crystal and the surface modifier [89]. This electron redistribution has the ability to modify both the electronic structure of the single-crystal surface and the active sites of the heterogeneous component. Consequently, this redistribution optimizes the adsorption and desorption energy of the active intermediates, ultimately leading to enhanced activity at the heterogeneous interface.

##### Nanoconfinement Effect

The nanoconfinement effect arises from the presence of nanoscale pores or spaces within the catalyst. When reactant molecules or particles are confined within these nano-sized spaces, it significantly affects their physical and chemical properties, thereby influencing their interaction with the catalyst surface [90]. Two-dimensional materials with atomic thickness can be applied to the surface of a single-crystal to create an atomic-molecular-sized gap at the heterogeneous interface. This enables the reactant molecules to enter via diffusion and undergo localized catalytic reactions on the surface of the single-crystal.

#### Classification of Single-Crystal Modification

##### Heterostructure Strategy

**Fig. 5:**
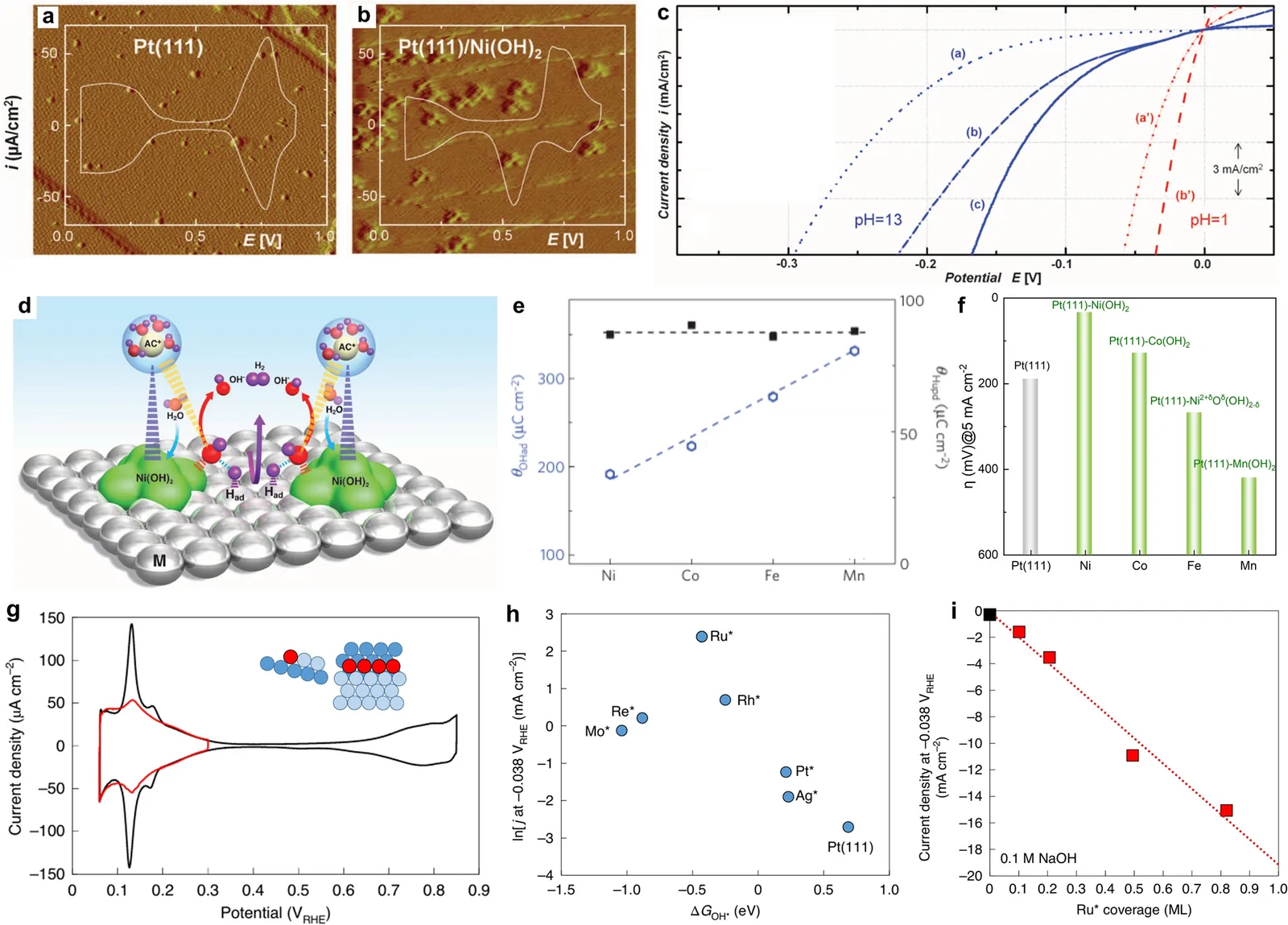
Behavior of H*/OH* on Pt(111) electrode modified with heterogeneous compounds. **a** STM images and CV of pristine Pt(111). **b** STM image and CV after modification with Ni(OH)_2_ clusters. **c** HER performances for Pt(111) and Ni(OH)_2_/Pt(111). **d** Corresponding dual-functional mechanism schematic. Reproduced with permission [97]. Copyright 2011, American Association for the Advancement of Science. **e** Universal trend of OH* charge versus metal cation oxophilicity. **f** HER overpotential of M(OH)_x_-Pt(111) (M = Ni, Co, Fe and Mn) at a current density of 5 mA cm^−2^ Reproduced with permission[91]. Copyright 2012, Springer Nature. **g** CVs of Pt(553) before and after Ru* decoration at step edges. **h** Relationship between HER activity ΔG_OH*_ on the M-modified surfaces (M = Ru, Rh, Ag etc.). **i** Relationship between HER activity and Ru coverage. Reproduced with permission[10]. Copyright 2020, Springer Nature

Constructing heterostructures on single-crystal surfaces has proven to be an effective strategy for enhancing electrocatalytic activity [86, 91–96]. However, the application of single-crystal surface modifications specifically for the HER has not been systematically reviewed. The importance of OH* is further demonstrated on single-crystals modified by oxyphilic metals. For instance, Markovic et al. prepared a Ni(OH)_2_/Pt(111) heterostructure. The STM clearly shows the random distribution of Ni(OH)_2_ clusters across the Pt(111) terraces (Fig. 5a, b). As shown in Fig. 5c, the HER overpotential of Ni(OH)_2_/Pt(111) is approximately 100 mV lower than that of Pt(111) (at a current density of 6 mA cm^−^^2^).The authors attributed the improvement in the HER performance to the bifunctional effect, namely that Ni(OH)_2_ promoted the dissociation of water and Pt sites promoted the generation of H_2_ from H* (Fig. 5d) [97]. While hydroxyl binding energy serves as a key descriptor for improving the alkaline HER kinetics, it must still adhere to the Sabatier principle. In the following year, the same group prepared 3d-transition metal hydr(oxy)oxide modified Pt(111) to establish the functional relationship between the strength of the OH-M^2^⁺^δ^ bond and the HER performance [91]. This relationship reveals that a clear trend of OH-M^2+δ^ bond energy strength (Ni < Co < Fe < Mn) is negatively correlated with the HER performance (Mn < Fe < Co < Ni), implying that the metal–OH bond strength is a primary factor governing activity enhancement. The adsorption strength and stability of OH* on Pt(111) can be fine-tuned by engineering its composition, structure, and surface properties, consequently optimizing the catalytic activity and selectivity **(**Fig. 5e, f).

The strategic passivation of specific high-index Pt(hkl) sites with heteroatoms serves dual purposes: i) It enables investigation of the bifunctional synergy between the single-crystal surface and the heteroatomic species; ii) it allows structure–activity relationships bridging low-index and high-index single-crystal configurations to be established. For example, Koper et al. used heteroatom site modification on the Pt(553) using the step modification method. When Ru atoms are modified at the step edges, the peak intensity of UPD_H_ decreases (Fig. 5g). Systematic investigation of metal species (Mo, Re, Ru, Rh, Ag) revealed a classic volcanic trend between the alkaline HER rate and the hydroxyl binding strength, highlighting the critical importance of achieving a moderate OH* adsorption energy for optimal performance (Fig. 5h). In addition, the relationship between metal coverage and HER activity has also been explored (Fig. 5i). Consequently, the HER kinetics are governed by a dual dependence on both the optimized H* adsorption rate on the Pt as well as the OH* desorption rate [10, 30, 41, 77].

**Fig. 6:**
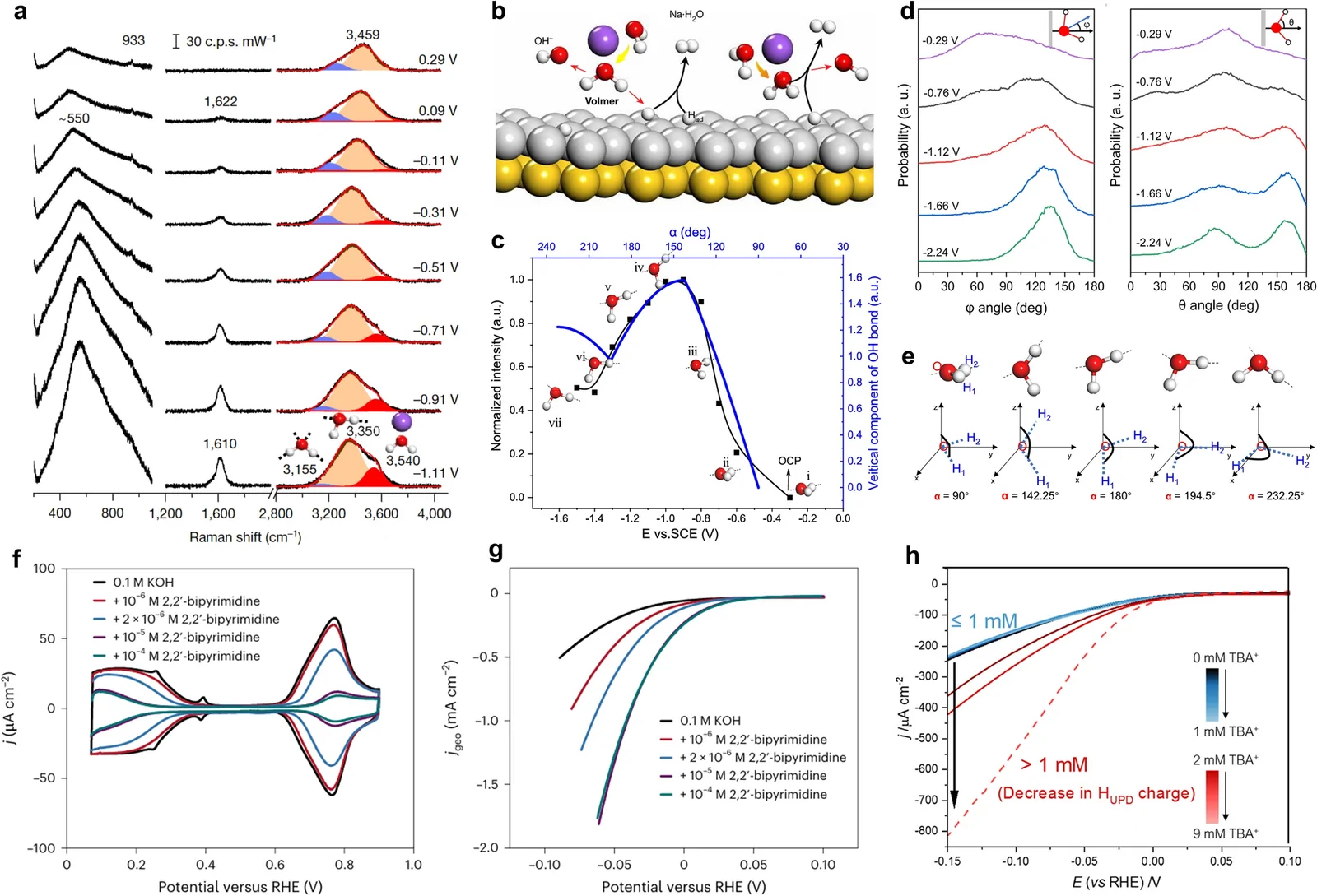
Probing the behavior of H_2_O* on modified Au(111) surfaces via in situ Raman spectroscopy and electrochemistry. **a** In situ Raman of H_2_O* on a Pd(111) in a 0.1 M NaClO_4_. **b** Schematic illustration of the interfacial behavior of Na^+^ and H_2_O*. Reproduced with permission[44]. Copyright 2022, Springer Nature. **c** Intensity changes of the OH stretching band of 2-HB H_2_O. The black axis represents the normalized intensity of the OH stretching band, and the blue axis represents the vertical component of the OH bond. **d** AIMD results showing the calculated distribution of the θ and φ angles. **e** Different configurations of a water molecule. Reproduced with permission [98]. Copyright 2023, Cell Press. **f** CVs of Pt(111) in Ar-saturated 0.1 M KOH electrolyte solutions containing different concentrations of 2,2′-bipyrimidine. **g** HER performance of Pt(111) in Ar-saturated KOH electrolyte solutions containing different concentrations of 2,2′-bipyrimidine. Reproduced with permission [99]. Copyright 2025, Springer Nature. **h** Linear sweep voltammograms of Pt(111) in pH12 NaOH containing TBA^+^ during the HER. Reproduced with permission [100]. Copyright 2024, American Chemical Society

The investigation of H_2_O* on heterostructured single-crystals presents both significant challenges and scientific appeal, as it involves complex interplay between water molecular structures and electronic structures at the atomic scale. Wang et al. conducted in situ SHINERS studies on the arrangement and dynamic evolution of water molecules at the interface of a Pd(111)/Au(111) electrode. Their investigation identified the presence of a group of cation-bonded water molecules (Na-H_2_O) at the electrode/electrolyte interface, in addition to the well-known hydrogen-bonded water molecules (Fig. 6a). An increased population of Na⁺-H_2_O species was observed at more negative potentials. These Na-H_2_O species reside closer to the electrode surface compared to hydrogen-bonded water molecules (Fig. 6b). Furthermore, elevating the electrolyte concentration is observed to augment the proportion of Na-H_2_O in the interfacial region, consequently enhancing the HER rate. They also identified that the electronic structures of the Pd impacts the quantity of Na-H_2_O and the rate of the HER, emphasizing the strong influence that Na-H_2_O has on promoting the HER rate [44].

Extending their research beyond metallic interfaces, Wang et al. investigated H_2_O* on a graphene/Au(111) heterostructure. They utilized the wet transfer method to transfer graphene onto an Au(111) surface. Changes in the Raman peak intensities of water indicated a gradual transformation in the orientation of H_2_O* molecules beneath graphene: from a parallel orientation to a single H-down orientation and eventually to a double H-down orientation as the electrode potential was shifted negatively (Fig. 6c). Additionally, AIMD simulations confirmed the structural transformation process of H_2_O* (Fig. 6d, e). These findings represent a significant advancement in comprehending the fundamental processes of water confined at the graphene interface and provide guidance for the design of efficient electrocatalytic interfacial surfaces [98].

##### Molecular or Ion Modification Strategy

A molecular modification strategy refers to the intentional adsorption or anchoring of organic molecules, coordination complexes, or molecular species onto an electrode surface (single-crystal or nano-catalyst surface) to modulate its electrocatalytic properties. The adsorption of molecules onto electrode surfaces constitutes a crucial area of investigation in single-crystal electrochemistry. Xu et al. reported a molecular design strategy to enhance the HER performance of Pt(111) by introducing a strongly adsorbed organic overlayer (Fig. 6f, g). 2,2'-Bipyrimidine specifically adsorbs on Pt(111), increasing the HER current density by approximately sixfold at −0.03 V vs. RHE. DFT calculations revealed that the HBE on the exposed Pt sites of the functionalized Pt(111) is weakened, attributed to the downward shift of the d-band center in the top Pt layer, which alleviates the over-binding of H* on the Pt surface. Importantly, the authors demonstrate the effectiveness of the organic overlayer in a water electrolyzer with a membrane electrode assembly configuration, employing Pt/C as the cathode catalyst and adding 2,2'-bipyrimidine in the catholyte, highlighting the practical implications of the proposed molecular functionalization strategy [99]. Although this work demonstrates an innovative molecular modification strategy for modulating HER activity on well-defined single-crystal surfaces, it does not provide direct spectroscopic evidence for intermediate species.

Beyond molecular modifiers such as bipyrimidine, organic cations have also been employed as a modification strategy to enhance the alkaline HER on Pt single-crystal electrodes, as demonstrated by Koper (Fig. 6h) [100]. TBA⁺ accumulates in the diffuse double layer and forms a reversible, two-dimensional physical adsorption film at concentrations exceeding 1 mM, and the reduction in the hydrogen region area in CV confirms this. TBA⁺ hydrophobic nature reorganizes the interfacial hydrogen-bond network, facilitating proton shuttling through the double layer and thereby accelerating the Volmer step. The enhancement is further amplified on stepped surfaces such as Pt(553), where step sites promote stronger TBA⁺ interactions, leading to HER improvement even at low concentrations. Collectively, this work illustrates that molecular or ion modification strategies need not rely on covalent bonding or direct electronic modulation of the catalyst; instead, physical adsorption organic cations can enhance HER activity by tailoring the interfacial microenvironment, including water structure and cation distribution, offering a complementary route to traditional heterostructure or defect engineering approaches. Organic compounds exhibit diverse functional groups and adjustable alkyl chain structures, rendering metal–organic interfacial catalysts a significant component of heterogeneous catalysis.

##### *Mechanisms of Performance Enhancement *via* Modification Strategy*

The enhanced HER performance of heterostructures constructed on single-crystal surfaces arises from several synergistic factors. First, the bifunctional effect enables cooperative catalysis, where one component promotes water dissociation in alkaline media, while the adjacent component facilitates H* recombination to form H_2_, thereby lowering the overall overpotential. Second, electronic structure modulation at the heterointerface induces charge transfer that shifts the d-band center of the metal catalyst, weakening H* binding energy to a more favorable level, as observed in Ni(OH)_2_/Pt(111). Third, heterostructures contribute to stabilization of key reaction intermediates, where the oxyphilic metal hydroxide component strongly binds OH* intermediates generated during water dissociation, preventing Pt site poisoning and preserving active sites for H* conversion to H_2_.

Molecular modification of single-crystal surfaces enhances HER performance through several interconnected mechanisms. First, modulation of the electronic structure of the metal catalyst is a primary factor. Adsorbed organic molecules, such as 2,2'-bipyrimidine on Pt(111), induce charge transfer that shifts the d-band center of the top Pt layer downward, thereby weakening the excessively strong HBE on Pt. This optimized H* adsorption strength facilitates H_2_ desorption and reduces the overpotential. Second, molecular modifiers can stabilize specific reaction intermediates through non-covalent interactions via their functional groups, thereby lowering the reaction barrier. Third, molecular modification alters the interfacial microenvironment, including hydrophobicity, local pH, ion distribution, and water molecule orientation at the electrode/electrolyte interface, which influences mass transport and interfacial reaction kinetics of H_2_O, H⁺, and OH⁻.

### Validated Insights from Single-Crystal Studies to Nano-Catalysts

This subsection explicitly summarized key findings from single-crystal investigations that have been successfully validated on nano-catalyst systems to establish a conceptual bridge single-crystal studies and nano-catalysts.

#### Structure–Activity Relationships of Single-Crystal Facets

**Fig. 7:**
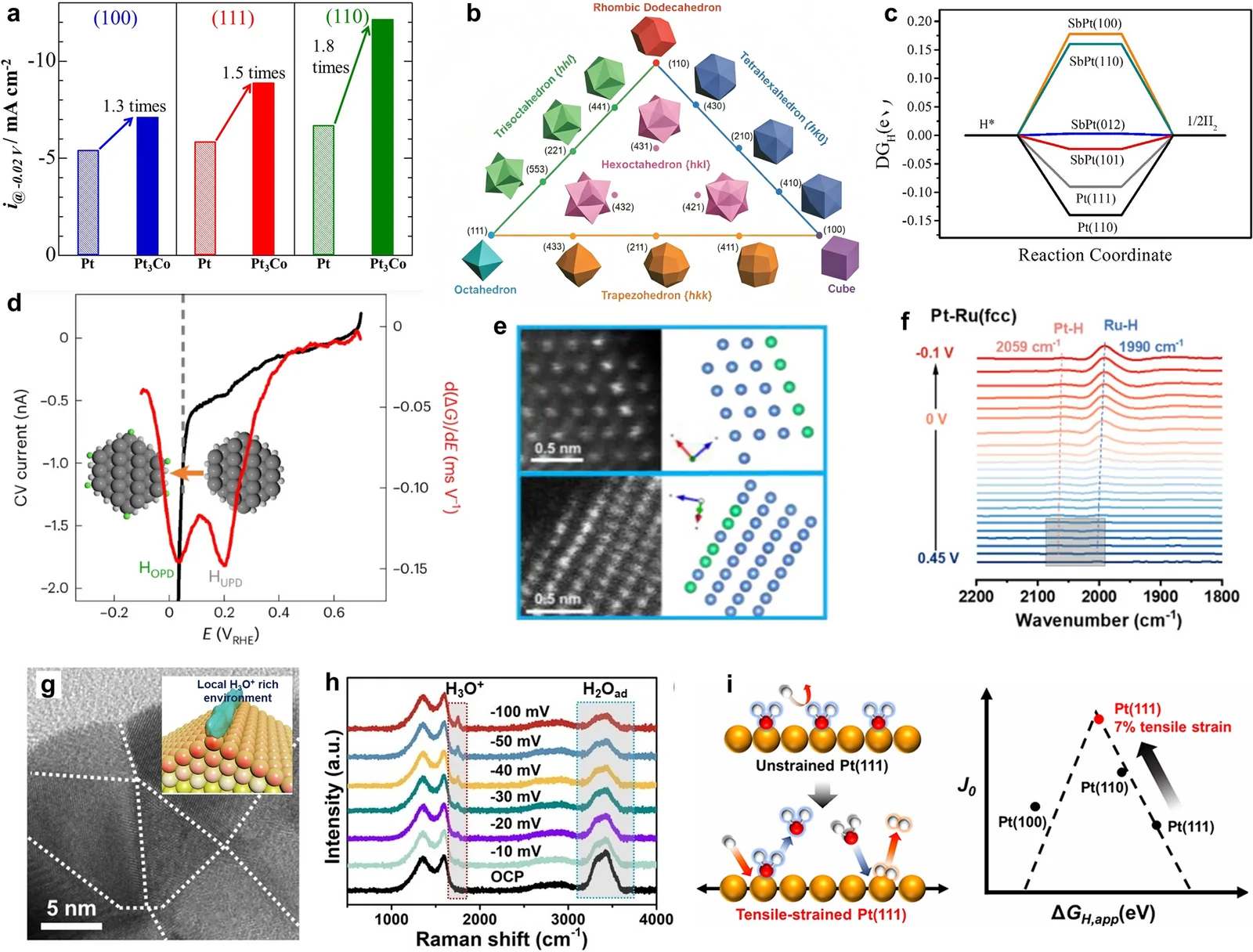
**a** Current densities for the HER measured at −0.02 V vs. RHE on Pt and Pt-skin/Pt_3_Co single-crystal electrodes with (111), (100), and (110). Reproduced with permission [101]. Copyright 2019, Elsevier. **b** Unit stereographic triangle of polyhedral nanocrystals enclosed by different types of crystalline facets. Reproduced with permission [105]. Copyright 2013, American Chemical Society. **c** Calculated free energy diagrams for H* adsorption on different crystal facets. Reproduced with permission [109]. Copyright 2021, Elsevier. **d** Derivative conductance changes and CV profile across the entire hydrogen adsorption region, revealing two distinct hydrogen adsorption peaks: the commonly observed UPD_H_ peak at 0.20 V vs. RHE and a newly identified OPD_H_ peak at 0.038 V vs. RHE. Reproduced with permission [33]. Copyright 2024, Springer Nature. **e** High-angle annular dark-field scanning transmission electron microscopy (HAADF-STEM) image of Pt-Ru pairs at the Pt-Ru heteroatomic interface. **f** In situ attenuated total reflection SEIRAS spectra of the H* on Pt-Ru. Reproduced with permission [113]. Copyright 2025, Wiley–VCH. **g** TEM images of Ir with rich grain boundaries. **h** Raman spectra of Ir with rich grain boundaries. Reproduced with permission [112]. Copyright 2024, Wiley–VCH. **i** Calculated free energy diagrams for H* adsorption on unstrained Pt(111) and tensile-strained Pt(111). Reproduced with permission [114]. Copyright 2026, American Chemical Society

As shown in Fig. 7a, classical single-crystal studies have established a clear hierarchy of HER activity on Pt surfaces, generally following the order Pt(110) > Pt(111) > Pt(100) [101]. This order has been rationalized by differences in the geometric arrangement of active sites. Critically, this facet-dependent activity hierarchy has been directly validated on shape-controlled Pt nanoparticles. Nanoparticles enriched with Pt(110) or high-index facets consistently exhibit markedly higher HER activity than those dominated by (111) or (100) facets, as demonstrated by rotating disk electrode measurements and theoretical calculation [102, 103].

Beyond the simple low-index facet hierarchy, single-crystal studies on stepped surfaces (e.g., Pt(553), Pt(533)) have demonstrated that high-index facets and stepped surfaces, which contain higher densities of undercoordinated sites, generally exhibit enhanced HER kinetics due to optimized H*/cations adsorption behaviors [36, 104]. The relative proportions of different crystal facets exposed on the surface of Pt nanocrystals can be precisely controlled by tailoring their morphology (Fig. 7b) [105]. High-index Pt facets, consistently outperform octahedral and cubic counterparts [106, 107]. Direct comparisons of turnover frequencies and Tafel slopes reveal that the enhancement magnitude correlates with the fraction of high-index surface sites, in quantitative agreement with single-crystal stepped surface studies [108]. For example, Chan et al. synthesized raspberry-like SbPt nanoparticles exposing high index (012) facets [109]. DFT calculations revealed that these facets possess lower adsorption energies than low index Pt facets, thereby modulating the HER pathway through optimized H* chemisorption (Fig. 7c). Consequently, the SbPt nanoparticles achieved superior HER activity, with a Tafel slope of 50.5 mV dec⁻^1^ and an overpotential of only 81 mV at 10 mA cm^−2^ in 0.5 M H_2_SO_4_. These validations provide compelling evidence that mechanistic insights from single-crystal electrodes are applicable to nanoscale electrocatalysts.

In summary, single-crystal studies have established a robust mechanistic foundation that is largely transferable to nano-catalyst systems. However, nano-catalysts introduce additional layers of complexity, including particle size effects, interparticle interactions, support effects, and dynamic restructuring.

#### Structural Complexity in Nano-Catalysts: Defects and Grain Boundaries

The key levels of complexity (defects, grain boundaries and strain, etc.) must be considered when extrapolating single-crystal data to nanoparticle systems [110]. Nanoparticles contain a non-negligible density of low-coordination sites at edges, corners, steps, and kinks. Recent advances in electrocatalysis have highlighted the crucial role of unsaturated surface sites in modulating the H* behavior of reaction intermediates and governing HER activity. Using a combination of electrical transport spectroscopy (ETS) and reactive calculations, Duan and co-workers have provided direct evidence that edge sites on Pt nanowires dominate the HER activity. These coordinatively unsaturated edge sites facilitate the adsorption of OPD_H_ at a potential of approximately 0.038 V vs. RHE, which is significantly closer to the HER equilibrium potential compared to the UPD_H_ adsorbed on terrace sites at 0.20 V vs. RHE (Fig. 7d). Consequently, hydrogen atoms bound to edge sites exhibit a moderate and near-optimal adsorption free energy, making them thermodynamically more favorable for HER. Moreover, in alkaline media, OPD_H_ formation on edge sites is markedly suppressed, which accounts for the much slower HER kinetics. These findings establish that unsaturated edge sites serve as the primary active centers in Pt-based HER catalysts by providing optimal HBE and facilitating efficient HER, thereby offering a critical mechanistic framework for the rational design of high-performance electrocatalysts [33].

In polycrystalline nanoparticles or electrochemically deposited films, grain boundaries represent two-dimensional defects where lattice misorientation occurs [111–113]. Zhao et al. constructed a Pt atomic chain-modified Ru cocrystalline structure featuring abundant Pt-Ru heteroatomic interfaces (Fig. 7e) [113]. Using in situ ATR‑SEIRAS and DFT calculations, they identified that the bridge‑adsorbed hydrogen intermediate on short‑range Pt-Ru pair sites dominates the HER (Fig. 7f). The strong electronic coupling between Pt and Ru at the interface facilitates electron transfer from the Pt-Ru pair to the adsorbed H_bridge_, leading to a moderate hydrogen adsorption free energy. Hou et al. synthesized grain boundary‑enriched Ir for alkaline HER (Fig. 7g) [112]. They demonstrated that grain boundary induces compressive lattice strain and reduce the coordination number of surface Ir atoms, which in turn generates an electron‑enriched surface. This electron‑rich environment promotes the formation of H_3_O^+^ intermediates at the grain boundary, creating a local acid‑like environment even under alkaline conditions (Fig. 7h). These H_3_O^+^ species lower the water dissociation energy barrier and supply sufficient protons to enable hydrogen spillover from grain boundary to adjacent terraces, thereby synergistically enhancing the HER, which is unattainable on flat single-crystal terraces. These defect studies bridge the gap between idealized surface science models and practical high-performance electrocatalysts, transforming the structural disorder once avoided in single-crystal studies into a powerful tool for modulating reaction pathways, local solvation environments, and synergistic spillover effect. Cho and colleagues investigate how tensile strain on Pt(111) surfaces affects the HER in acids. The authors synthesized unstrained Pt octahedra and tensile-strained Pt icosahedra (~ 7% lattice expansion). As a result, the strained icosahedra achieved a 1.3-fold higher exchange current density than unstrained octahedra, demonstrating that lattice strain improves HER kinetics by modulating the competitive adsorption between hydrogen and interfacial water (Fig. 7i) [114].

## HER on Polycrystalline Nano-Catalysts

Inspired by the well-defined mechanistic principles established on single-crystal surfaces, it is now essential to examine how these insights translate to structurally complex nano-catalysts. The structure of electrocatalysts governs the behavior of reactive intermediates and ultimately dictates the HER activity [115, 116]. Understanding intermediate behavior on such realistic, non-idealized surfaces is therefore critical for bridging the gap between fundamental mechanistic insights derived from model systems and the design of high-performance, practical HER catalysts. While numerous reviews have comprehensively addressed the structural characterization of HER catalysts [89, 117], this review focuses specifically on intermediate species and their characterization methodologies during HER processes.

### Active Species Involved in the HER on Polycrystalline Catalysts

#### Hydrogen Adsorption (H*)

**Fig. 8:**
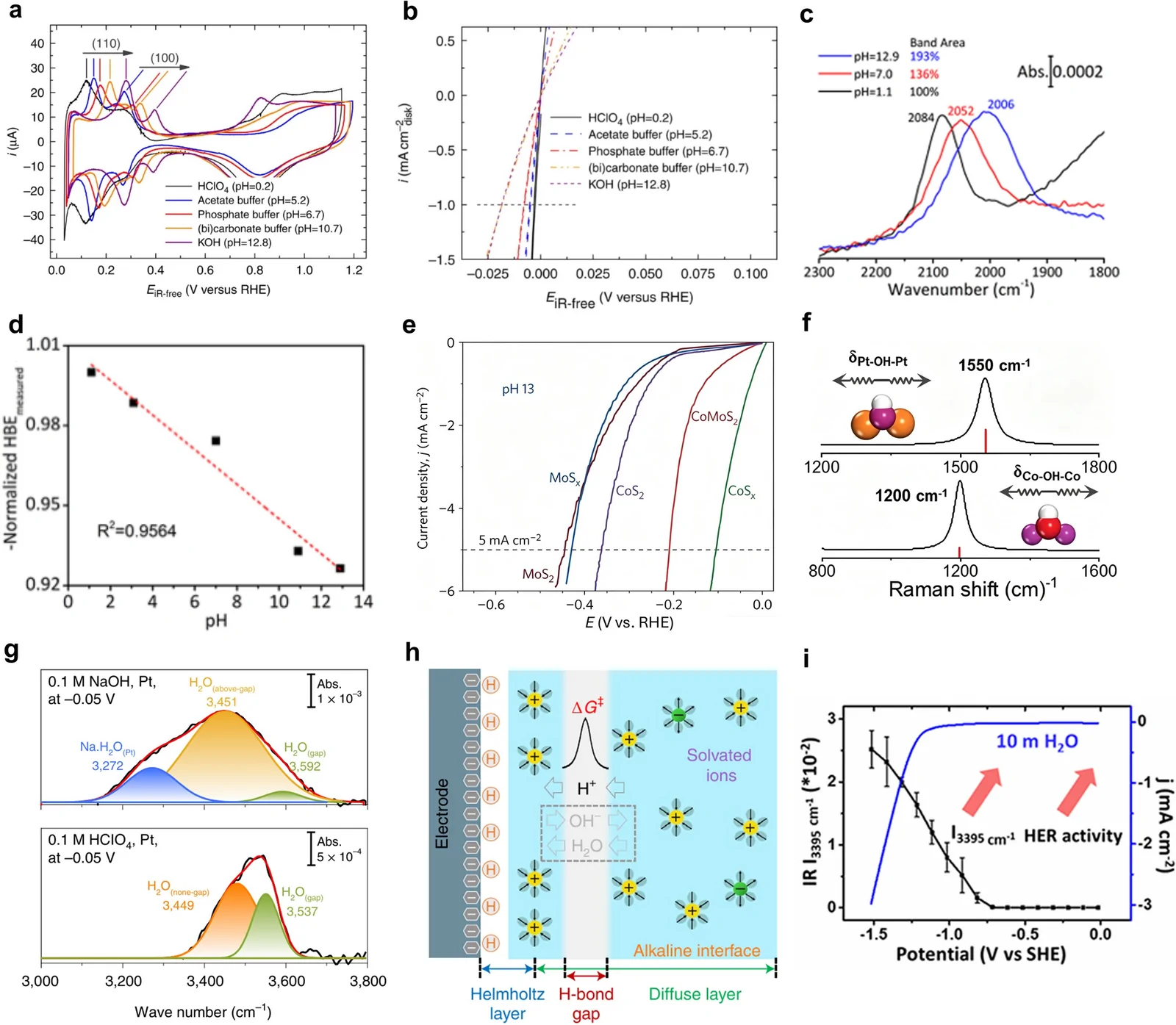
Research of H*, OH* and H_2_O* on polycrystal electrode. **a** CVs of Pt measured in selected Ar-saturated electrolytes. **b** HER polarization curves of Pt collected in selected H_2_-saturated buffered electrolytes. Reproduced with permission [11]. Copyright 2015, Springer Nature. **c** Quantitative comparison of the Pt–H bands recorded on Pt in Ar-saturated 0.1 M HClO_4_ (black), phosphate-buffered saline (PBS) (red), and NaOH (blue) at 0 V. **d** Derived correlation between the normalized HBE and solution pH. Reproduced with permission [29]. Copyright 2020, American Chemical Society. **e** HER polarization curve of ultrafine nanoalloys with different metal compositions. Reproduced with permission [121]. Copyright 2016, Springer Nature. **f** Raman peaks for δ_Co-OH-Co_ and δ_Pt-OH-Pt_, respectively. Reproduced with permission [122]. Copyright 2024, Wiley–VCH. **g** Experimental O–H stretching vibration profiles recorded at −0.05 V vs. RHE in NaOH and HClO_4_. **h** A structural depiction of the EDL is presented. Reproduced with permission [28]. Copyright 2022, Springer Nature. **i** Integrated intensity of the ν(O–H) vibrational signature at 3395 cm⁻^1^ is correlated with the HER current density [127]. Copyright 2023, American Chemical Society

Yan et al. investigated the correlation between the HBE on polycrystalline Pt and the electrolyte pH using electrochemical methods (Fig. 8a). Results showed that in the potential range of 0.0 to 0.5 V vs. RHE, the UPD_H_ characteristic peaks on polycrystalline Pt originated from adsorption on Pt(110) and Pt(100) sites. As the pH increased from 0.2 to 12.8, the potential of the peaks shifted positively by approximately 0.15 V, indicating a significantly enhanced HBE on Pt surfaces under alkaline conditions [11]. This trend suggests that the positive shift in peak potential, reflecting a stronger HBE on Pt, correlates with a decrease in HER activity as pH increases (Fig. 8b). This phenomenon elevates the activation energy barrier of the HER, providing a mechanistic explanation for the sluggish HER kinetics at high pH. Similar pH-dependent HER activity patterns were validated in Ir, Pd, and Rh nano-catalysts [118]. However, in situ spectroscopic evidence for the pH dependence of HBE exhibited contrasting results: HBE was observed to decrease with increasing pH, a discrepancy potentially arising from the distinct natures of UPD_H_ and OPD_H_ [29]. Shao et al. used ATR-SEIRAS to investigate the pH-dependency of H* binding energy on Pt surfaces, quantifying monotonically weakened HBE with increasing pH (Fig. 8c, d), which may represent a critical factor for the reduced HER activity. Further studies revealed that HBE is a synergistic outcome of electric field effects, H* coverage, and altered Pt–water/H*–water interactions [29]. The authors subsequently explored the contributions of Pt and Ru in the HER: on pristine Pt and Ru-modified Pt surfaces. Pt–H spectral peaks showed negligible shifts, indicating that Ru atoms do not significantly alter the electronic properties of uncovered Pt surfaces. In contrast, Ru–H peaks on Ru-modified Pt shifted to higher wavenumbers compared to pure Ru, suggesting enhanced binding of hydrogen intermediates. This implies that Ru modification induces strain and electronic effects in Pt, lowering the Volmer step energy barrier as the rate-determining step [30]. Complementing these findings, Han et al. demonstrated that the lower energy barrier for H* spillover from Ru to Ni sites, as revealed by comparative analysis of migration free energy, indicates a synergistic catalytic mechanism. This process is further corroborated by Ru K-edge variations at different potentials which correlate with H_2_O adsorption and dissociation, while Ni K-edge changes indicate its primary role in the Tafel step of H* dimerization to form H_2_. ΔG_H*_ values show near-thermoneutral conditions at Ni sites, favoring H* dimerization to form H_2_[119].

In summary, the pH dependence of HBE on Pt and related catalysts remains a subject of debate, with different experimental approaches yielding contrasting trends. Electrochemical studies on polycrystalline Pt reveal that UPD_H_ characteristic peaks shift positively by approximately 0.15 V as pH increases from 0.2 to 12.8, indicating significantly enhanced HBE under alkaline conditions, which explains sluggish alkaline HER kinetics. However, in situ ATR-SEIRAS studies by Shao et al. show the opposite trend that weakened HBE with increasing pH, suggesting that the discrepancy may arise from the distinct natures of UPD_H_ and OPD_H_. Further investigations indicate that HBE may a synergistic outcome of electric field effects, H* coverage and H*–water interactions.

#### OH Adsorption (OH*)

Building on studies of oxophilic metal modification on single-crystal surfaces, Markovic et al. extended this concept to practical catalyst systems, demonstrating that Ir and Pt_0.1_Ru_0.9_ alloys exhibit significantly higher HER/HOR activity than Pt in alkaline solutions, attributed to stronger OH* adsorption on oxophilic sites (surface defects on Ir and Ru atoms in PtRu alloys) [120]. This strategy was extended to non-noble electrocatalysts, where the CoMoS_x_ hybrid exhibited high activity and stability across both acidic and alkaline media (Fig. 8e). The observed kinetics were attributed to a critical balance between the water dissociation rate and the OH^δ−^ desorption rate [121]. Zhou et al*.* further validated the importance of appropriate OH* adsorption energy and proposed a gradient desorption model for OH*. Ultrasmall five-element PtCoCuNiZn nanoalloys exhibited exceptional OH* desorption capability in the alkaline HER, with an OH* desorption potential (0.6 V) 40 mV higher than PtCoCuZn (0.56 V), indicating easier OH* desorption. In situ Raman spectroscopy revealed unique dual-site synergism: peaks at ~ 1200 and 1550 cm^−1^ corresponded to OH vibrations on Co and Pt sites, respectively, whereas control materials (PtCoCuZn, PtCuZn, etc.) showed only Pt site peaks (Fig. 8f). This discrepancy was attributed to the gradient desorption model of the five-element alloy, where strongly adsorbed OH undergoes stepwise desorption via bimetallic sites, thereby optimizing OH* adsorption strength and promoting water dissociation. Combined machine learning simulations and experiments confirmed that this mechanism significantly enhances the alkaline HER activity, offering new insights for designing efficient electrocatalysts [122].

In summary, OH* plays a multifaceted role in HER. On oxophilic-modified surfaces such as Ni(OH)_2_/Pt(111), OH* acts as a direct participant in the bifunctional mechanism by promoting water dissociation leading to better HER performance. However, when OH* is too strongly adsorbed, it becomes a poison by blocking active H* sites and reducing activity. Moreover, OH* interacts intimately with the hydrogen-bond network of interfacial water, where structure-making cations such as Li⁺ stabilize ordered water networks that facilitate OH* desorption and proton transfer. Thus, the effect of OH* is guided surface structure, oxophilic modification, and electrolyte composition.

#### ***H***_***2***_O* Structure (H_***2***_***O)***

H_2_O* research has established itself as a fundamental area of electrocatalysis [123–125]. Interfacial H_2_O* studies on nano-catalysts are pivotal due to their unique structural complexity and decisive role in reaction mechanisms. Unlike idealized single-crystal surfaces, nano-catalysts exhibit heterogeneous active sites, such as facets, edges, and defects that create diverse local aqueous environments. This heterogeneity results in non‑uniform H_2_O* orientation, hydrogen‑bonding networks, and solvation structures, which directly modulate key steps in electrocatalytic reactions, including water dissociation and proton electron transfer. Therefore, elucidating the behavior of interfacial H_2_O* on nano-catalysts is essential not only for understanding activity origins and selectivity but also for guiding the rational design of catalysts optimized for real‑world operating conditions.

Chen et al. investigated H_2_O* networks under different pH conditions using SEIRAS (Fig. 8g). They demonstrated that ordered and interconnected H_2_O* networks in acidic environments promote the HER kinetics, whereas alkaline conditions give rise to a “water-deficient gap” leading to fragmented hydrogen-bonding networks (Fig. 8h). This structural discontinuity hinders proton and OH⁻ transport, thereby explaining the diminished HER performance in alkaline media [28]. Building on the understanding of H_2_O*, Jia et al. demonstrated that introducing N-methylimidazole (Me-N_1_C_2_) at the Pt-H_2_O interface significantly enhances the HER performance in alkaline media [126]. They proposed that Me-N_1_C_2_ stabilizes the second hydration layer to facilitate interfacial OH^−^ diffusion, thereby enhancing the HER/HOR kinetics. Based on these findings, a universal mechanism was established: The HER/HOR kinetics on Pt surfaces are controlled by H⁺ diffusion rates within the interfacial hydrogen-bonded networks in acidic media and by OH⁻ diffusion rates in alkaline media, respectively. This study concluded that charged Pt-water interfaces disrupt hydrogen-bonding networks and hinder ion diffusion, whereas molecular modification to restore interfacial hydrogen bonding represents an effective strategy to optimize the HER/HOR kinetics. To further explore H_2_O* engineering, Jiang et al. designed a nanoreactor system and discovered that hydrophilic Li⁺ ions promote water dissociation at short ranges but disrupt water network connectivity at long ranges, while 1-butyl-3-methylimidazolium tetra fluoroborate ionic liquids ([BMIM][BF_4_] ILs) promote the formation of extended asymmetric four-coordinate networks at the interface (Fig. 8i). This structure enhances OH⁻ transport during the Volmer step within the EDL, thereby boosting the HER activity. These results highlight that the connectivity of the hydrogen-bonded network controls optimal water dissociation and charge transport for maximum HER performance [127].

**Fig. 9:**
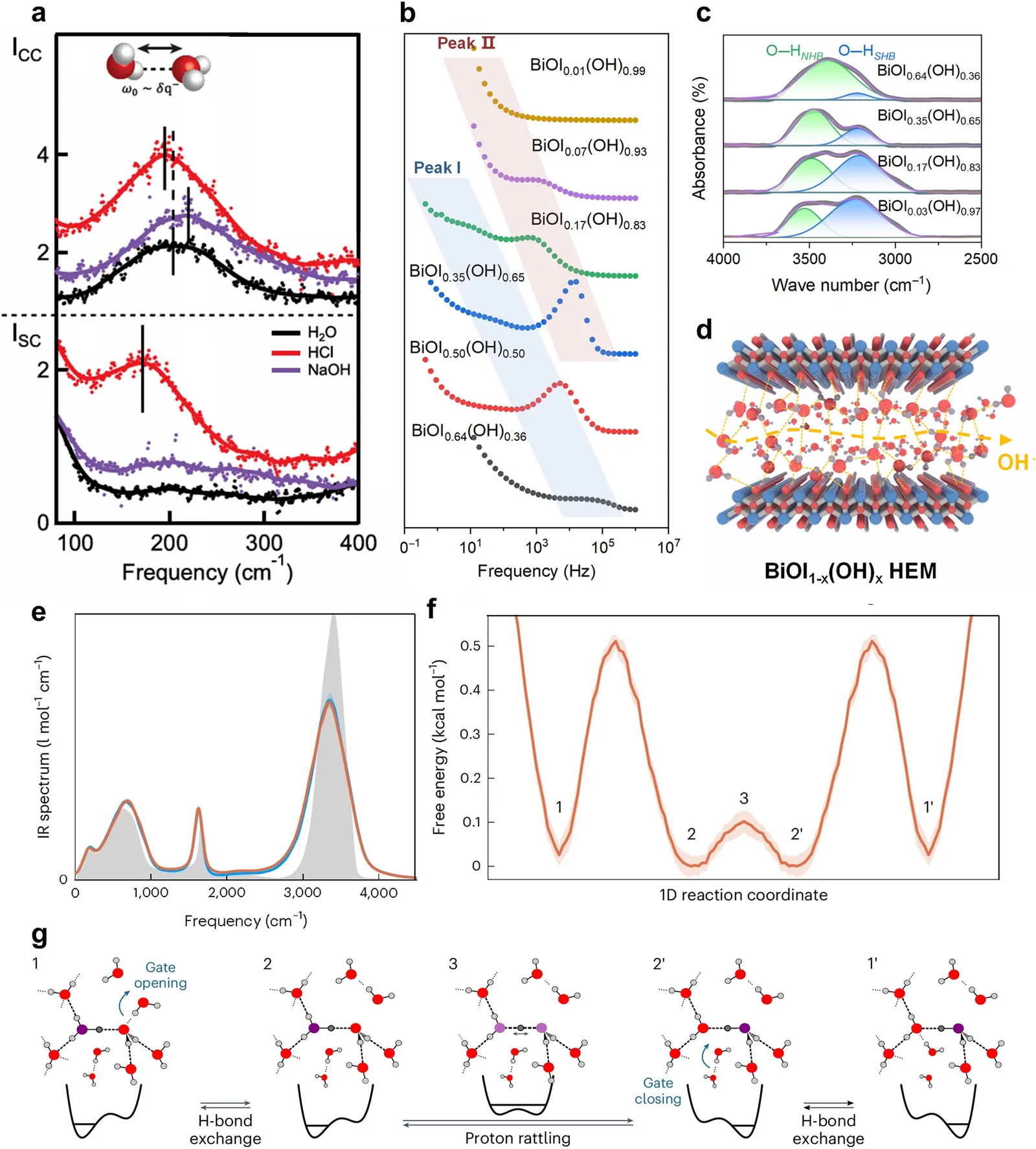
H_2_O* dynamics in polycrystalline catalytic systems. **a** Self-correlation (ICC) and cross-correlation (ISC) low-frequency vibrational spectra. Reproduced with permission [128]. Copyright 2024, American Association for the Advancement of Science. **b** Dielectric response of hydroxide-substituted BiOI_1-x_(OH)_x_. **c** Raman spectra of BiOI_1-x_(OH)_x_. **d** Proposed mechanism for OH⁻ transport within the nanoconfined space. Reproduced with permission [129]. Copyright 2025, American Association for the Advancement of Science. **e** Calculated IR spectra of the acidic (orange) and neat water (blue) solutions, with the experimental spectrum (gray) of liquid water. **f** Free energy profile along the pathway. **g** Schematic proton transfer mechanism with typical H-bond arrangements and proton potential energy profiles for key locations on the surface. Reproduced with permission [136]. Copyright 2024, Springer Nature

Consequently, the role of hydrogen-bonding networks in mass transport has attracted significant research interest. Roke et al. detected vibrational and hydrogen bond stretching modes via terahertz and Raman spectroscopy, as well as two-dimensional infrared and Raman-terahertz echo measurements. By analyzing hydrogen bond stretching (~ 200 cm^−1^) and bending (~ 70 cm^−1^) vibrations in water (Fig. 9a), they demonstrated that charge transfer and non-quadratic effects (NQEs) can be deconvoluted. Studies reveal that acidic solutions exhibit a higher density of NQEs than alkaline solutions, which facilitates more efficient charge transfer [128]. As the Grotthuss mechanism governs hydroxide and proton transport in hydrogen bonding networks, Zhou et al. proposed an innovative two-dimensional confinement strategy. With hydroxide substitution, the peak I shifts down to 8.68 Hz, and the slower dielectric relaxation indicates the formation of more hydrogen bonds (Fig. 9b). The constructed nanochannels by (bismuth oxyiodide (BiOI) nanosheets induce dense short hydrogen bond (SHB) networks within narrow interlayer spaces (Fig. 9c). By tuning interlayer spacing and introducing hydroxyl groups to enhance hydrophobicity, the number of SHBs in BiOI channels increased by approximately 12-fold, drastically promoting Grotthuss-type OH⁻ diffusion. The material also exhibited exceptional performance and durability in water electrolyzers. Dielectric spectroscopy, infrared spectroscopy, low-field NMR, and simulations collectively confirmed that SHB formation increased significantly with hydroxyl substitution rates, primarily originating from confined water. The schematic diagram of OH diffusion through the corresponding confined space is shown in Fig. 9d [129]. Gomez used neural network-based molecular dynamics simulations and vibrational spectroscopy to reveal the mechanism of excess proton transport in water (Fig. 9e). They found that the hydrated proton exists in two stable hydrogen-bond arrangements (Eigen - and Zundel-like). Proton transport follows a multi-step sequential mechanism, and the free energy barrier for each step was calculated (Fig. 9f, g): First, a hydrogen bond breaks on the acceptor water, enabling proton transfer; second, the proton rapidly transfers across a low barrier; and third, a hydrogen bond reforms on the donor water. The mechanism explains key experimental observations, including the low diffusion activation energy and the long-lived Zundel-like species seen in ultrafast spectroscopy.

An alternative perspective suggests that disrupting the interfacial hydrogen-bonding network of water can be beneficial for HER activity. This disrupted hydrogen-bonding network of water increased molecular disorder and the formation of localized, non-uniform water clusters. Such alterations often arise from strong interactions with the catalyst surface (e.g., specific adsorption, electric fields), the presence of ions, or nanoconfinement effects. Xu et al. demonstrated that specifically adsorbed organic additives (theophylline derivatives) can enhance the intrinsic HER activity of polycrystalline Pt-based catalysts. The optimal HER activity was achieved on Pt surfaces modified with 7-n-butyltheophylline, attributed to the disruption of hydrogen-bonding networks in the EDL [43]. Complementing this approach, Wu et al. combined in situ characterization and electrochemical analysis to elucidate the correlation between dynamic evolution of H_2_O* and electrocatalytic performance across broad current density ranges. The basis of this H_2_O* modulation strategy lies in the interaction between reaction sites and water molecules under HER potentials. For example, in Pt@Ni(OH)_2_-NF, an electrochemical potential-induced phase transition of Ni(OH)_2_ disrupts the original rigid hydrogen-bonded network, thereby inducing the structural evolution of H_2_O* [6].

Some studies, based on vibrational in situ SEIRAS, report a predominance of water molecules with a “ice-like” or tetrahedrally coordinated structure (with a strong O–H stretch around 3200 cm⁻^1^), which is proposed to facilitate proton transfer through a Grotthuss hopping mechanism due to its well-ordered hydrogen-bonding network. while others, using SERS and SHINERS, emphasize a more disordered structure (with a dominant band near 3400–3600 cm⁻^1^), which is thought to promote HER by providing more flexible and reorientable water molecules to the electrode surface. These opposing conclusions are not necessarily contradictory but rather reflect different adsorption regimes, surface conditions, and experimental sensitivities.

**Fig. 10:**
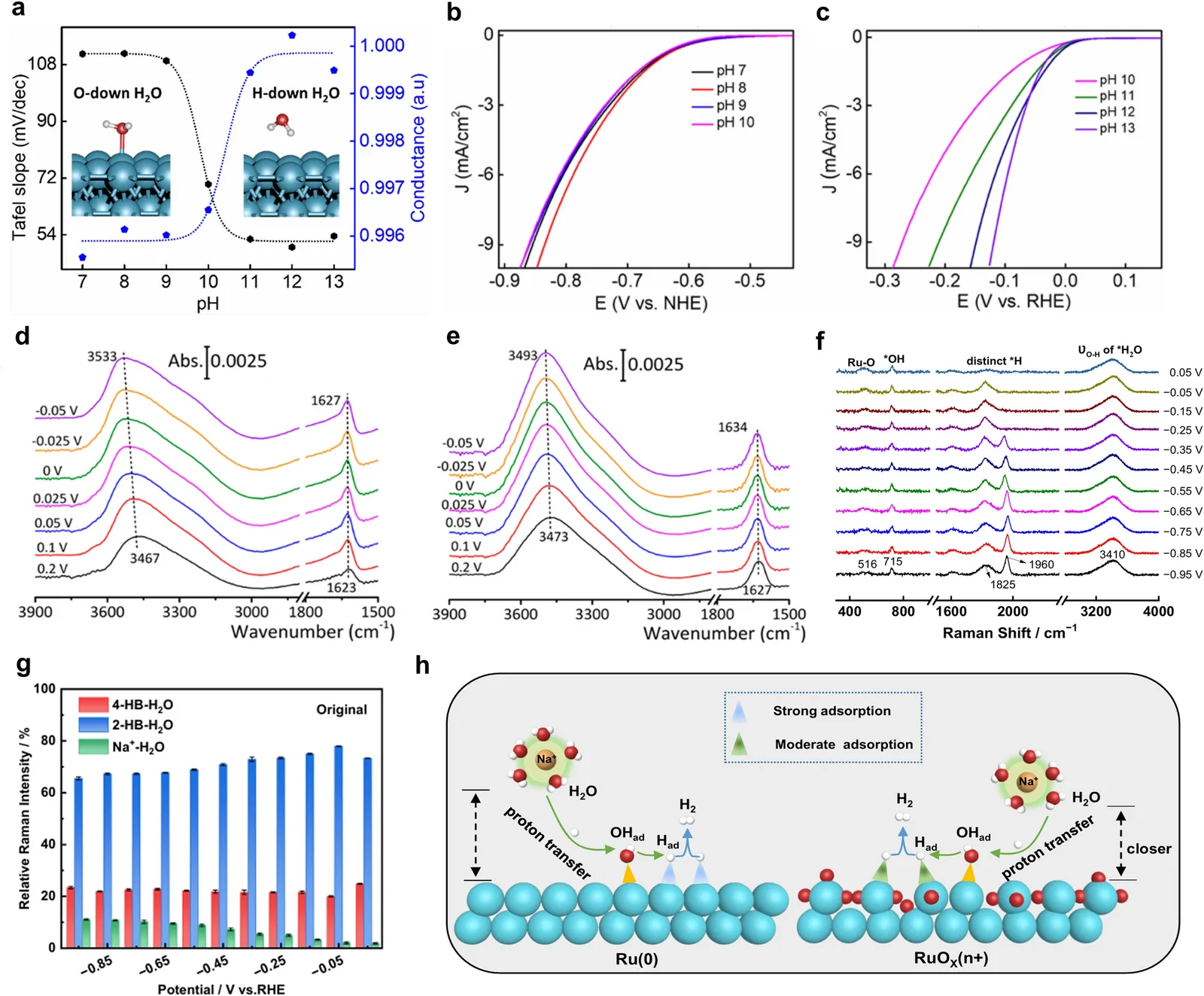
Behavior of H_2_O* on polycrystalline catalysts. **a** Electrical transport spectroscopy (ETS) measurements and structural models of water orientations on Pt(111) in low-pH and high-pH conditions. **b, c** HER activities at different pH. Reproduced with permission [130]. Copyright 2024, American Chemical Society. In situ ATR-SEIRAS spectra of the Pt working electrode recorded using applied potentials from 0.2 to −0.05 V in Ar-saturated 0.1 M **d** HClO_4_ and **e** NaOH electrolyte solutions. Reproduced with permission [29]. Copyright 2020, American Chemical Society. In situ Raman spectra of the HER at **f** Au@Ru in 0.1 M NaOH. **g** Normalized Raman intensities of νO-H on original Ru surfaces measured using applied HER potentials. **h** HER mechanism at Ru surfaces. Reproduced with permission [8]. Copyright 2023, Springer Nature

The specific orientation of interfacial water molecules governs key interfacial behaviors and reaction pathways, making its characterization an indispensable aspect of electrochemical interface research. Sun et al. used in situ electrochemical Raman spectroscopy to directly correlate H_2_O* structures with HER activity. They revealed that as solution pH increased, the structure of the first water layer on catalyst surfaces transitioned from acting predominantly as proton acceptors to acting as proton donors. By optimizing the electronic properties of PtNi catalysts, they demonstrated that under alkaline conditions, H_2_O* rearranged with more H atoms oriented toward the catalyst surface, thereby enhancing the HER performance [6]. Duan et al. further investigated orientation effects in their report on Pt surface properties under alkaline conditions using CV and electrical transport spectroscopy (ETS). They observed Tafel slopes of ~ 110 mV dec⁻^1^ at pH 7–10 and ~ 53 mV dec⁻^1^ at pH 11–13, indicating improved HER kinetics at higher pH (Fig. 10a). ETS revealed similar pH-dependent trends with a significant change near pH 10, suggesting significant changes in surface adsorbates. Fixed-potential calculations and chemical bond analyses attributed this phenomenon to shifts in H_2_O* orientation: Water molecules adopted an O-down configuration below pH 10 and an H-down configuration above pH 10. This reorientation weakened O–H bonds in H_2_O*, thereby accelerating the HER kinetics at higher pH (Fig. 10b, c) [130].

The structure, dynamics, and hydrogen-bonding network of interfacial water molecules directly influence the stability, adsorption configuration, and conversion kinetics of these key HER intermediates (H* and OH*). Therefore, a comprehensive mechanistic understanding of the HER, demands moving beyond the isolated study of adsorbed H_2_O* or intermediates to explicitly address their dynamic interactions between H_2_O* and intermediates. Shao et al. used SEIRAS to obtain experimental evidence of pH-dependent hydrogen-water binding strength: In alkaline media, vibrational peaks of H_2_O* at 3580–3450 and 3000 cm⁻^1^ decreased in intensity, indicating reduced water binding energy (Fig. 10d, e). These findings collectively reveal a competitive mechanism between water molecules and H* at interfaces, providing a new theoretical framework for interpreting the pH-dependent HER activity [29]. Li et al. used SERS to investigate HER mechanisms on Ru surfaces. They revealed that the modified electronic structures in RuO_x_, which are associated with higher Na·H_2_O content (Fig. 10f, g), favor moderate H* and OH* adsorption. This was further confirmed by the direct observation of two distinct H* vibrational modes (1825 and 1960 cm^−1^) on Ru^(0/n+)^ surfaces with different valences, along with direct visualization of OH* and H_2_O* adsorption during the alkaline HER [8]. This synergy between electronic modulation and solvation structure ultimately accelerates the alkaline HER kinetics by optimizing the H* and OH* adsorption pathways (Fig. 10h). Based on these insights they developed an Au@Ni(OH)_2_ catalyst to dynamically visualize the impact of interactions between H_2_O* and OH* on the HER. They found that OH* and water interactions promoted the formation of water with weak hydrogen bonds, creating a favorable environment for the HER. Additionally, deprotonation steps during the HER on Au@Ni(OH)_2_ were observed, confirming OH* participation in the reactions. Raman and X-ray photoelectron spectroscopy validated the phase transition of Ni(OH)_2_ to NiO, during which significant red shifts in OH* stretching frequencies of water molecules confirmed that surface OH* disrupted the interfacial hydrogen-bonded water network [7].

In summary, interfacial H_2_O* structure, orientation, and hydrogen-bonding networks critically determine HER kinetics, with effects highly sensitive to pH, potential, cation identity and surface structure. Ordered H_2_O networks in acidic media promote Grotthuss mechanism, while alkaline conditions create fragmented networks that hinder ion transport and slow HER. Opposing views suggests that disordered water offers flexibility that also promotes HER. Thus, precise control over H_2_O networks is essential for optimizing HER performance.

## Research Methods and Performance Improvement Strategies for Intermediate Species in Nano-Catalysts

As shown in Table 2, the study of intermediate species in the HER on nano-catalysts has evolved into a methodological framework characterized by the integration of multi-scale characterization with computational simulation, and equal emphasis on interfacial microenvironment and dynamic evolution. In terms of research methodology, in situ spectroscopic techniques (e.g., in situ SHINERS, FTIR and XAS) are predominantly employed to directly monitor the adsorption configurations and coverage changes of key intermediates such as H*, OH*, and H_2_O* under operating conditions.

**Table 2:** Literature reports discussing intermediate species in the HER published during the past three years

Catalysts	Overpotential	Electrolyte	Catalyst performance optimization strategy	The focused intermediates	Research methods for intermediates
PtCoCuNiZn [122]	10 mA cm⁻^2^@−33 mV	1.0 M KOH	The five-component alloy leads to the gradient desorption of OH*	OH*	Raman/DFT calculations
La_1_-Ru_n_/NC [137]	10 mA cm⁻^2^@100 mV	1.0 M KOH	Heterogeneous single-atom moderate OH* adsorption strength	OH*	Raman/DFT calculations
Ru/Ni(OH)_2_ [138]	100 mA cm⁻^2^@71 mV	1.0 M KOH	Benefiting from the heterostructure, the OH ion forms a stronger hydrogen bond interaction with the H_2_O* by acting as the electron-donor	OH⁻ /H_2_O*	XPS/Raman/DFT
Pt_1_@POMs@PC [88]	10 mA cm⁻^2^@less than 10 mV	0.5 M H_2_SO_4_	The nanoconfinement environment around Pt sites promotes H* spillover	H*	Raman/EIS/Theoretical calculations
RuNi/NC [119]	10 mA cm⁻^2^@ 12 mV	1.0 M KOH	Superhydrophilic and high-curvature carbon nanocages are anchored with pointed bimetallic RuNi nano-alloys	H*	XAFS/DFT calculations
Ru/Nb_2_O_5_ [139]	10 mA cm⁻^2^@ 31 mV	1.0 M PBS	Benefiting from the heterostructure, Nb_2_O_5_ can reduce the d-band center of Ru in Ru NCs	H_2_O*	Raman/AIMD
(Rh, O)-MoSe_2-x_ [140]	10 mA cm⁻^2^@ 40 mV	0.5 M H_2_SO_4_	Synergistic M–O dual-atom pairs	H_2_O*	Raman/AIMD
Pt island film [127]	-	[BMIM][BF_4_]ILs Ionic liquid	-	H_2_O*	SEIRA spectra/MD
RuSe_x_-RuNC [32]	10 mA cm⁻^2^@ 29 mV	1.0 M KPi	Benefiting from the heterostructure, RuSe_x_ disrupts the hydrogen bond network in the interface, thereby increasing the amount of H_2_O near RuNC	H_2_O*	SEIRAS
BiOI_1−x_(OH)_x_ Hydroxide exchange membranes (HEMs) Devices [129]	1760 mA·cm⁻^2^@2.0 V	15% KOH	Two-dimensional confinement strategy was employed, which utilized BiOI (bismuth iodide) nanosheets to construct nanochannels and induce the formation of dense short hydrogen bond networks in the narrow interlayer space	OH⁻ /H_2_O*	Dielectric spectra/FT-IR/AIMD
Coₛ-SO-Ru [141]	10 mA cm⁻^2^ @6 mV	0.1 M KOH	The three-dimensional hydrogen bond network constructed by the sulfur-oxygen bridge (-S = O···H_2_O) promotes the transport of protons from the solution to the interface through the “Grotthuss mechanism.”	H*/OH⁻ /H_2_O*	ATR-SEIRAS/DFT calculations
Pt/cage [142]	10 mA cm⁻^2^ @32 mV	0.1 M KOH	The interaction between H_2_O* and the -NH groups of the Pt cage frame softens the hydrogen bond network, making it more conducive to charge transfer	H_2_O*	SERS
Pt_1_Co_6_/np-Co_2_P [143]	10 mA cm⁻^2^ @21 mV	1.0 M KOH	Pt_1_Co_6_/np-Co_2_P significantly increases the concentration of K^+^ in the near-surface region. This improvement, in turn, enhances the Pt–H bond and promotes H-OH bond dissociation and promotes the dissociation of H-OH bonds	H_2_O*	ATR-SEIRAS/SERS

## Summary and Outlook

In this review, the intrinsic mechanisms of the HER from the perspective of key reactive intermediate species have been discussed in detail. By integrating in situ spectroscopic characterizations (e.g., in situ Raman, FTIR) and electrochemical testing techniques, an in-depth summary and analysis of the interfacial behaviors of critical intermediates such as H*, OH* and H_2_O* has been provided, revealing the dynamic behaviors of the intermediates in acidic and alkaline media. These research advances have not only facilitated the development of novel electrocatalysts, but more significantly, have provided fundamental new insights into catalytic mechanisms. Despite the significant breakthroughs in nano-catalysts design and the wealth of interfacial process information obtained via advanced characterization techniques, a unified theoretical framework for HER mechanisms remains incomplete. Future research must combine state-of-the-art in situ characterization with theoretical calculations to elucidate the adsorption-conversion mechanisms of intermediates across model systems, from single-crystals to practical nano-catalysts. This endeavor will represent a key challenge in achieving precise regulation of hydrogen electrocatalysis. To conclude, the following sections provide prospective areas for further development.

### Development of In Situ Techniques

The continuous development of novel in situ techniques is indispensable for fundamentally advancing our mechanistic comprehension of intermediate species in the HER. While established in situ methods have provided invaluable insights, the inherent complexity and transient nature of the electrochemical interface demand tools with ever-greater sensitivity, specificity, and spatiotemporal resolution. Next-generation in situ methodologies, such as time-resolved vibrational spectroscopy with ultrafast lasers, and cryo-electron microscopy for electrochemical systems are poised to overcome current limitations. These advancements will enable the direct visualization of short-lived intermediates, the mapping of potential-dependent intermediate coverages with atomic precision, and the differentiation of chemically similar species (e.g., atop vs. bridge-bonded H*) on complex, nanostructured catalysts.

### Precise Characterization of HER Intermediates via Multi-Technique Synergy

The accurate characterization of intermediate species in the HER necessitates the integration and cross-validation of multiple analytical techniques. A synergistic approach combining complementary in situ and operando spectroscopies, such as Raman/IR for vibrational fingerprints. However, inherent ambiguities in spectral interpretation, potential-induced artifacts, and surface sensitivity limitations mean that findings from one technique must be rigorously validated by others. Furthermore, computational simulations play an indispensable role in refining experimental assignments by predicting vibrational modes, adsorption geometries, and coverage-dependent shifts. Only through such a concerted, multi-technique framework, where results are continuously cross-referenced and iteratively refined, can the community develop a reliable, atomic-scale understanding of HER intermediates and their role in governing catalytic activity and mechanism.

### A Reaction Cell Operating under Conditions Closer to Industrial-Scale Systems

Conventional electrochemical measurements on model single-crystals and in situ characterizations are typically conducted at ambient temperature and pressure. This default condition often overlooks the significant influence of temperature and pressure on electrocatalytic processes. According to the Nernst equation, temperature and pressure are two important factors affecting electrode potential. In reactions involving gaseous reactants or products, the pressure in the reactor affects dissolution equilibria, adsorption equilibria, and consequently the electrode potential. Therefore, developing in situ techniques capable of operating under controlled, variable temperature and pressure, mimicking industrial operating conditions (e.g., elevated temperatures in PEM electrolyzers), is vital.

### Advanced Computation and Artificial intelligence (AI) in Understanding HER Intermediates

The introduction of more advanced theoretical calculations and AI is poised to fundamentally transform the characterization and understanding of intermediate species in the HER. First, high-throughput AIMD simulations, accelerated by machine learning interatomic potentials (MLIPs), will allow researchers to model complex nano-catalyst surfaces with explicit solvation under operational electric fields. Second, AI and machine learning will act as powerful integrators and predictors. By training on vast datasets combining computational descriptors (e.g., d-band center, charge distribution) and multi-modal experimental results (from spectroscopy, microscopy, and electrochemistry), AI models can uncover structure–property relationships. They will not only predict the adsorption energies and vibrational signatures of intermediates on novel materials with high speed but also reverse-engineer optimal catalyst structures by identifying the key interfacial descriptors that govern intermediate stability and reaction kinetics.

### Deconvolution and Assignment of Interfacial Water Peaks

Beyond these condition-dependent differences, the assignment and deconvolution of the interfacial water peaks still require further experimental clarification. To address the broader issue, we propose a simple framework based on three key factors: the applied potential, temperature, and the type of electrolyte. Within this framework, we can identify three distinct water adsorption regimes. The first regime is ice-like water (~ 3200 cm⁻^1^). A simple test to confirm this regime is to warm the system, which will cause the ice-like structure to melt and the peak to disappear. The second regime is liquid-like water. It appears at negative potentials, which is exactly the range where the HER occurs. Here, the electrode surface carries a significant negative charge, which pulls water molecules into a different orientation (hydrogen atoms pointing toward the surface). This disrupts the ordered network, making the water behave more like bulk liquid (~ 3400 cm⁻^1^). The third regime involves water interacting strongly with cations. These cations accumulate near the negatively charged surface and bring their surrounding water molecules with them. The assignment of cation-coordinated water can be unambiguously achieved by systematically tuning the electrolyte cation concentration and identity. Specifically, increasing the cation concentration enhances the intensity of the split peak near 3550 cm⁻^1^, while replacing larger cations with smaller ones shifts the peak position and alters its splitting pattern due to differences in hydration shell rigidity and cation–water interaction strength. Such cation-dependent spectral fingerprints provide a direct and reliable means to deconvolute the contribution of cation-coordinated water from other interfacial water species, thereby enabling a more precise understanding of how cation hydration influences the EDL.

Our framework provides a clear and practical way to distinguish each regime and to interpret experimental results. Nevertheless, as noted above, the precise assignment and deconvolution of the interfacial water peaks remain an open challenge that will require further experimental efforts.

### From Nano-Catalysts to Practical Applications

While the preceding sections have provided mechanistic insights into the roles of H*, OH*, and interfacial water structure based primarily on studies using rotating disk electrode (RDE) setups and single-crystal surfaces at relatively low current densities, we acknowledge that practical electrocatalytic devices for hydrogen production operate under substantially different conditions. Gas diffusion electrodes (GDEs) and membrane electrode assemblies (MEAs) are designed to achieve high current densities by overcoming mass transport limitations inherent to conventional RDE configurations.

Several key challenges complicate this translation. First, mass transport limitations are minimal in RDE setups due to forced convection, whereas GDEs rely on a three-phase interface. At high current densities, local depletion of reactants and accumulation of products (e.g., OH⁻ in alkaline HER or H_2_ bubbles) can create substantial concentration gradients that are absent in RDE experiments. Second, local pH at the catalyst surface can deviate dramatically from the bulk electrolyte under high current densities. For the HER in neutral or weakly buffered media, local alkalinization near the cathode can shift the effective pH by several units, potentially altering the dominant reaction mechanism and the role of OH* and interfacial water. Third, the elevated temperatures often employed in industrial electrolyzers further modify water structure, ion mobility, and reaction kinetics in ways not captured by room-temperature single-crystal studies.

To bridge this gap, we highlight several emerging strategies. Operando characterization techniques adapted for GDE systems, including confocal Raman spectroscopy and infrared thermography, are beginning to provide spatially resolved information on local pH, temperature, and intermediate speciation under high-current–density operation. Additionally, the design and modification of in-situ working condition equipment offers intermediate platforms that maintain well-defined mass transport while accessing current densities closer to practical devices.

In conclusion, we emphasize that while single-crystal/nano-catalysts in situ spectroscopy provides an essential mechanistic foundation, direct extrapolation to device-relevant conditions must be undertaken with caution. Future work should prioritize correlative studies that combine well-defined model systems with operando characterization of GDEs under high-current–density operation, enabling the rational design of electrolytes and catalysts that perform optimally under practical conditions.
